# Alternative Approach to Sequence-Specific Recognition of DNA: Cooperative Stacking of Dication Dimers—Sensitivity to Compound Curvature, Aromatic Structure, and DNA Sequence

**DOI:** 10.1021/acschembio.4c00800

**Published:** 2025-02-07

**Authors:** Ananya Paul, J. Ross Terrell, Abdelbasset A. Farahat, Edwin N. Ogbonna, Arvind Kumar, David W. Boykin, Stephen Neidle, W. David Wilson

**Affiliations:** † Department of Chemistry and Center for Diagnostics and Therapeutics Georgia State University, Atlanta, Georgia 30303, United States; ‡ Department of Pharmaceutical Organic Chemistry, Faculty of Pharmacy, Mansoura University, Mansoura 35516, Egypt; § Master of Pharmaceutical Sciences Program, California North State University, Elk Grove, California 95757, United States; ∥ School of Pharmacy, University College London, London WC1N 1AX, U.K.

## Abstract

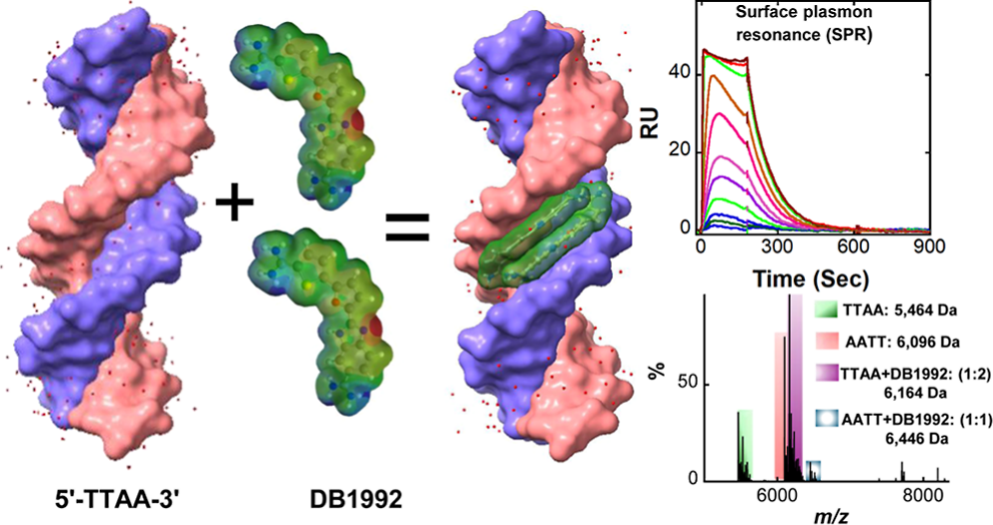

With the growing number and diversity of known genome sequences, there is an increasing opportunity to regulate gene expression through synthetic, cell-permeable small molecules. Enhancing the DNA sequence recognition abilities of minor groove compounds has the potential to broaden their therapeutic applications with significant implications for areas such as modulating transcription factor activity. While various classes of minor groove binding agents can selectively identify pure AT and mixed AT and GC base pair(s) containing sequences, there remains a lack of compounds capable of distinguishing between different AT sequences. In this work, we report on the design compounds that exhibit selective binding to -TTAA- or -TATA- containing DNA minor groove sequences compared with other AT ones. Several studies have shown that the -AATT- and -TTAA- sequences have distinct physical and interaction properties, especially in terms of their different requirements for recognition in the minor groove. Achieving strong, selective minor groove binding at -TTAA- sequences has been challenging, but DB1003, a benzimidazole–furan–furan diamidine, has demonstrated cooperative dimeric binding activity at -TTAA-. It has significantly less binding preference for AATT. To better understand and modify the selectivity, we synthesized a set of rationally designed analogs of DB1003 by altering the position of the five-membered heterocyclic structure. Binding affinities and stoichiometries obtained from biosensor-surface plasmon resonance experiments show that DB1992, a benzimidazolefuran–thiophene diamidine, binds strongly to -TTAA- as a positive cooperative dimer with high cooperativity. The high-resolution crystal structure of the TTAA–DNA–DB1992 complex reveals that DB1992 binds as an antiparallel π-stacked dimer with numerous diverse contacts to the DNA minor groove. This distinctive binding arrangement and the properties of diamidines at the -TTAA- minor groove demonstrate that benzimidazole–furan–thiophene is a unique DNA binding pharmacophore. Competition mass spectroscopy and circular dichroism studies confirmed the binding stoichiometry and selectivity preference of the compounds for the -TTAA- sequence.

## Introduction

The ability to target specific nucleic acid sequences and conformers with small molecules has a prominent role in both biotechnology and drug discovery research.^1−7^ Such specific binding compounds show promise to assume a major role in many areas including determination of disease states,^8−12^ drug targeting of gene expression,^13−16^ transcription factors,^17−19^ antifungal and antimycobacterial agents,^20−22^ transposon insertion,^23,24^ RNA in viral genomes,^25−28^ and bacterial translation.^29−32^ Our laboratories have been particularly interested in AT-specific DNA minor groove binding compounds.^33−41^ Research on these types of compounds began over 50 years ago with the polyamide natural products, netropsin, and distamycin, as well as synthetic diamidine compounds such as pentamidine and berenil.^42−47^ Initially the synthetic compounds were composed of *N*-methyl pyrrole and *N*-methyl imidazole, as with distamycin and netropsin, but groups interested in therapeutic applications of minor groove binders have extended the heterocyclic library. The groups at the University of Strathclyde, for example, have included a range of substituents, such as thiazole, substituted phenyl, and larger aromatic substituents, with a variety of cationic groups.^3,20,21^ They now have compounds with promising cellular activity against some very potent microbial parasites.^48,49^ The Lown, Wemmer, Dervan, Lee, Sugiyama, and Bashkin groups have also expanded on pyrrole and imidazole with additional heterocycles to develop polyamides with activity against a number of types of cancer.^50−59^ However, several factors have limited the medicinal/biological use of polyamides and those compounds have not been used in human clinical trials.^58−61^ A wide variety of synthetic heterocyclic amidines have also been designed, studied with DNA, and evaluated for biological activity by the Spanish groups of Mascarenas and Vaquez.^15,62^ Particularly important are their linked compounds and agents with attached functional groups based on DNA-targeted proteins.^19,63,64^ In parallel research, the SteidL group has shown excellent uptake into human cells by heterocyclic diamidines with low toxicity and promising activity against acute myeloid leukemia.^17,65^ They have also performed extensive genomic studies to show that the compounds can displace targeted transcription factors from cellular gene promoters to explain their anticancer properties and abilities to modify gene expression. Starting with the earliest tests of therapeutic applications of diverse minor groove binders, diamidine compounds have shown excellent cell uptake with clinical activity against a wide range of parasitic diseases.^2,17,18,34,65−67^

**Scheme 1:**
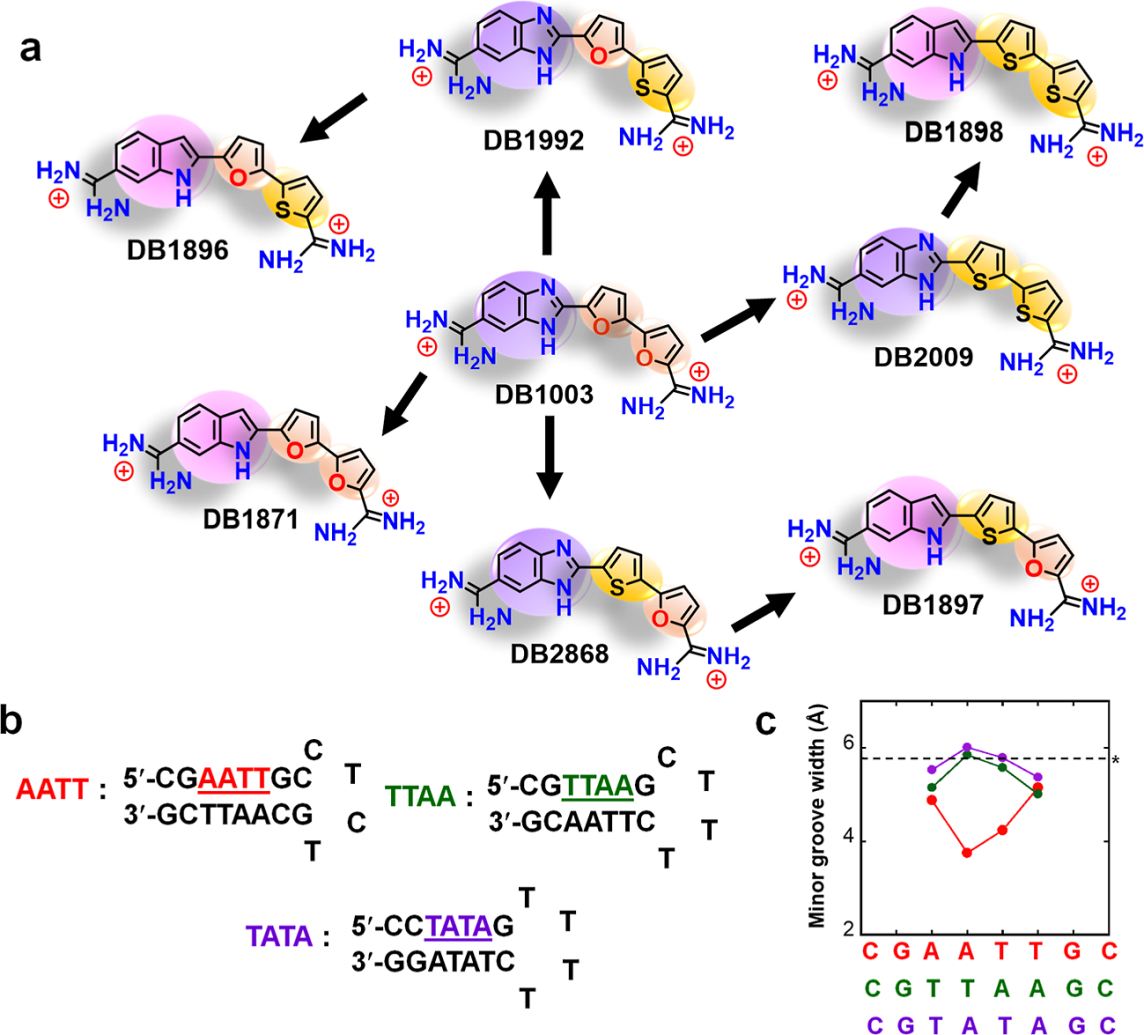
(a) Chemical Structures of Heterocyclic Diamidine Compounds Used in This Study; Color Schemes Used in This Figure Denote Different Functional Groups. (b) The Oligomeric (DNA) Sequences Used in This Study; DNA Sequences Used for Surface Plasmon Resonance (SPR) Studies Were Labeled with 5′-Biotin. To Evaluate Their Stability Oligomeric Sequences, Thermal Melting Temperatures (*T*_m_) of -AATT-, -TTAA-, and -TATA- Have Been Measured. (c) Minor Groove Width vs Target DNA Sequences Calculated from the Online Algorithm of Rohs and Co-workers (*Nucleic Acids Res.***2013,***4,* W56–W62) Thermal melting experiments were performed on a UV–vis spectrophotometer. The concentration of each hairpin DNA sequence was 3 μM, and experiments were in TNE100 buffer. The used oligomeric sequences are highly stable, with *T*_m_ values of 71 °C for -AATT-, 59 °C for -TTAA-, and 58 °C for -TATA- (Figure S1). *The dashed line indicates the minor groove width of standard *B*-form DNA. Groove width is defined as the perpendicular separation of helix strands drawn through the closest complementary phosphate groups, minus 5.8 Å, to account for van der Waals radii of phosphate groups.

Given the successful use of AT sequence-specific heterocyclic diamidines in human and animal cells,^17,18,66,67^ our laboratories began an integrated synthetic and biophysical search for compounds to recognize mixed sequences of DNA. We have now prepared an array of compounds capable of recognizing mixed sequences of DNA with both AT and GC bp.^68−76^ Interestingly, although heterocyclic diamidines with appropriate shape, functional groups, and curvature can bind strongly to -AATT- sites, as in the original crystal structures, they generally bind to the reverse sequence, -TTAA-, more weakly.^77−81^ Given the optimized minor groove for binding monomeric compounds, such as polyamides and heterocyclic diamidines in AATT sequences, the generally weak binding to -TTAA- is apparently due to the higher malleability of the groove at -TTAA-, which can give a wider minor groove in the -TTAA- sequence (Scheme 1),^82^ as found in several crystal structures containing this sequence.^83−86^ As disclosed below, this TTAA groove is easily widened to accommodate a pair of stacked ligands. The malleability of the -TTAA- groove apparently gives weaker interactions with water and a wider groove on average than with -AATT-, as will be described below.

Heterocyclic compounds with the appropriate shape can stack with the walls of the minor groove in -AATT- and related sequences, but the wider groove in -TTAA- and -TATA- sequences reduces the stacking energetics. Both the -TTAA- and -TATA- sequences are very important in the human genome.^23,24,80,81^ The -TATA- box consensus sequence promoter DNA and TATA box binding protein, for example, is required in the initiation of eukaryotic gene transcription.^80^ The sequence is required for the different types of RNA transcription by all three RNA polymerases.^80^ The -TATA- DNA sequence thus occupies a critically important role in DNA biology. The isomeric-TTAA- sequence also plays an important biological role. The piggyBac transposon superfamily has been recently recognized because the piggyBac transposon has a broad host spectrum from yeast to mammals. This mobile element has been widely used for a variety of applications in a diverse range of organisms.^23,24^ The insertion recognition sequence for the transposon is -TTAA-, and after insertion, the inserted sequence has -TTAA- at both 5′ and 3′ ends. Because of the importance of these -TTAA- and -TATA- sequences, the design of small molecules that can strongly and selectively bind to them is an important research priority.

We initiated a design search for synthetic compounds that could efficiently and selectively bind to -TTAA- and -TATA-. We have found that compounds with a benzimidazole (BI) or indole (In) linked with two furans could form stacked dimers to effectively bind to -TTAA- compared to -AATT-.^77,78^ These compounds are the first examples of heterocyclic dications that can form dimers to selectively recognize the DNA minor groove. This observation raises the question of whether the difuran module has favorable properties for dimer stacking and binding to the -TTAA- sequence and whether other five-membered heterocycles, such as thiophene, can also form a stacked dimer with effective binding to -TTAA-? To answer this question, we have synthesized all possible combinations of furan and thiophene diamidines with either benzimidazole or indole groups (Scheme 1).

The interactions of these compounds with duplexes containing either -AATT- or -TTAA–/–TATA- sites have been investigated by SPR, circular dichroism (CD), mass spectrometry (MS), and molecular dynamics (MD) simulations. We have also determined the crystal structure of DB1992, a diamidine with a heterocyclic core of benzimidazole–furan–thiophene, bound as a tetracationic dimer to a -TTAA- site. Surprisingly, the combined results clearly show that the compounds with two five-membered-ring systems have an absolute requirement for a central furan in order to form a favorable stacked dimer with -TTAA- and -TATA- sequences. We see the opposite result with monomer compounds binding to the -AATT- minor groove, where compounds with a central thiophene have the strongest binding.^88,89^ The crystal structure and MD analysis provide a structural explanation for this observation.

The ability to bind specific sequences with noncovalent stacked dimer compounds opens the possibility of cooperative interactions of small, cell-permeable compounds to interact more strongly with specific DNA sequences.^2,17,18,34,36,65,67,89,90^ Using noncovalent monomers to selectively bind to DNA sequences as dimers can optimize cell uptake while showing high affinity to the target sequences.

## Results

### SPR Binding Affinity Results for the -AATT- Binding Site

**Figure 1:**
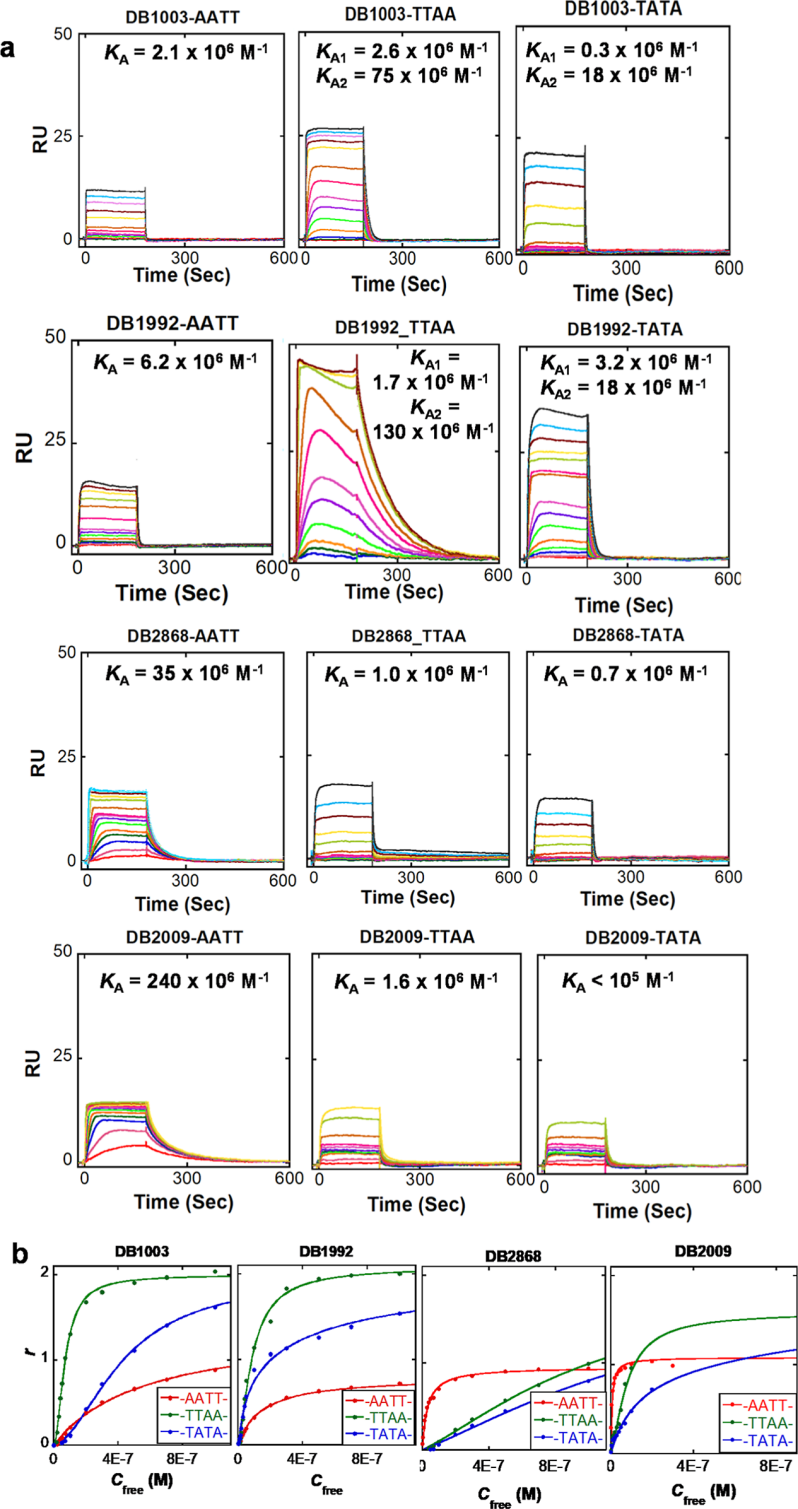
Effect of heterocyclic diamidines stacking at DNA minor grooves with different groove widths: (A) SPR sensorgrams for the interaction of DNA sequences (AATT, TTAA, and TATA) binding with DB1003, DB1992, DB2868, and DB2009. The injected concentrations of each ligand are 2, 5, 10, 15, 20, 30, 40, 50, 70, 100, 200, 300, 500, and 1000 nM in TNE 100 (50 mM Tris-HCl, 100 mM NaCl, 1 mM EDTA) buffer, pH 7.4. (B) Comparison of the SPR binding affinity for DNA sequences with some selected diamidines. RU values from the steady-state region of SPR sensorgrams are converted to *r* (*r* = RU/RU_max_) and are plotted against the unbound compound concentration (flow solution). The lines are the best-fit values of single- or two-site interaction models, and *K* values are given in Table 1.

The interactions of the heterocyclic diamidines (Scheme 1a) were investigated with three hairpin duplex sequences with -AATT-, -TTAA-, and -TATA-binding sites (Scheme 1b). To carry out this analysis, SPR detection was used, which enabled us to examine the binding of these compounds to immobilized DNAs on a biosensor surface. The biosensor-SPR method has the advantage of comparing binding affinities and stoichiometry to different DNAs since the same compound solution flows over the set of immobilized DNAs, yielding sensorgram saturation levels that can be compared directly for kinetics and stoichiometry differences.^91,92^ Sample signals at increasing concentrations of the compounds were observed to determine the binding constants for all of the heterocyclic diamidine derivatives depicted in Scheme 1. As expected, the original BI–furan–furan diamidine compound, DB1003, and other derivatives and isomers were observed to bind with the -AATT- sequence as a 1:1 complex.^93^ The compounds with a BI/In–thiophene–thiophene show high binding affinity for AATT sequences, as observed with DB818.^87^ The dithiophenes, DB2009 and DB1898 have the highest binding affinities with the -AATT- binding site (Figure 1 and Table 1). We obtained excellent sensorgrams for DB2009 and DB1898 at low concentrations with the -AATT- sequence, enabling kinetic fitting with a one-site model. The kinetic fittings of DB2009 and DB1898 with the -AATT- sequence resulted in findings of slow rates of association (*k*_a_ = 3.4 × 10^6^ M^–1^ s^–1^ for DB2009 and *k*_a_ = 1.3 × 10^6^ M^–1^ s^–1^ for DB1898) and dissociation (*k*_d_ = 11.9 × 10^–3^ s^–1^ for DB2009 and *k*_d_ = 5.9 × 10^–3^ s^–1^ for DB1898). Figure S3a shows these compounds to be robust -AATT- binders with an equilibrium binding constant *K*_A_ of 2.4 × 10^8^ M^–1^ for DB2009 and 1.5 × 10^8^ M^–1^ for DB1898 (Figure 1, Table 1, and Figure S3b).

**Table 1:**
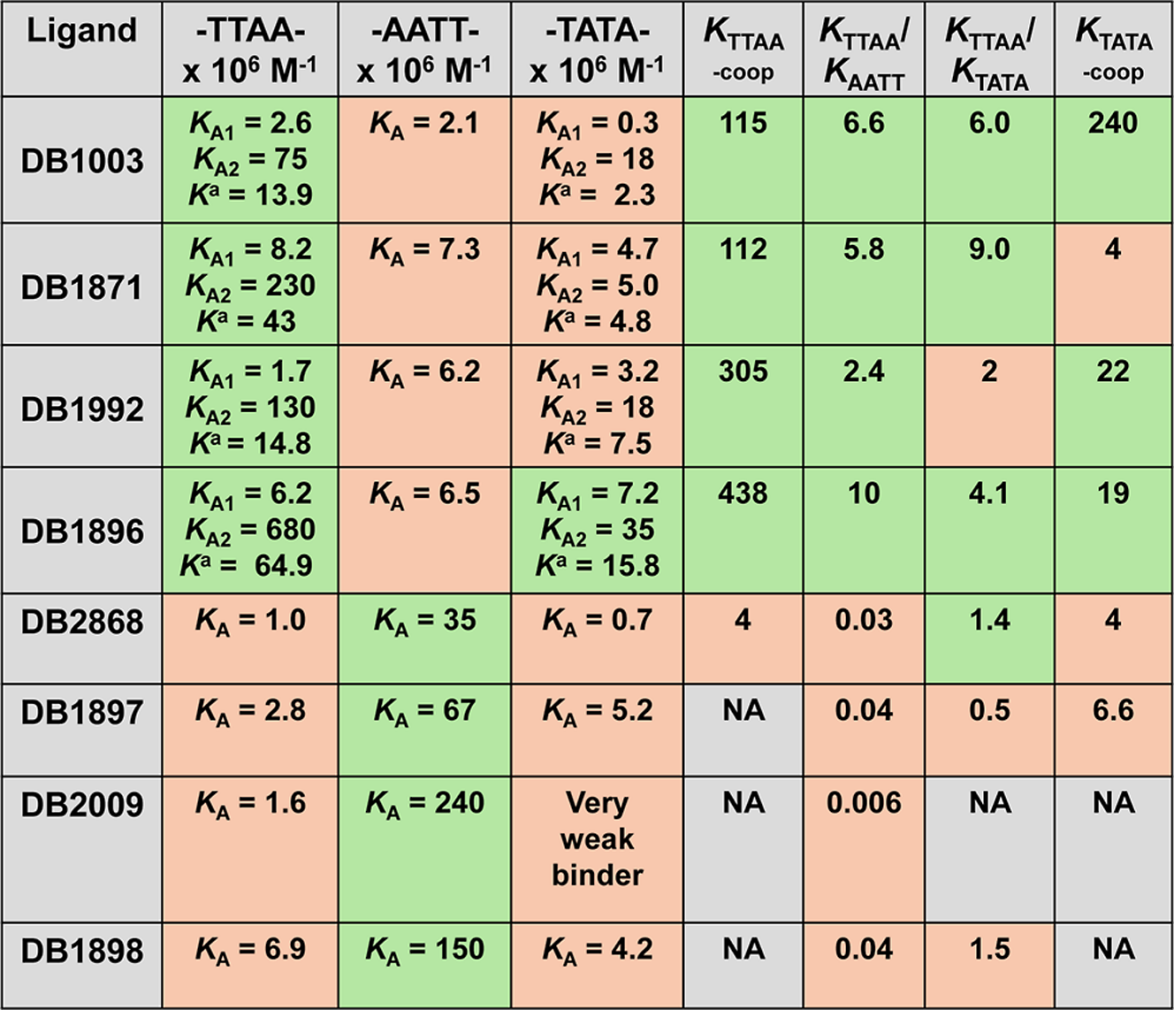
Summary of Equilibrium Binding Constant (*K*_A_ M^–1^) for the Binding Interaction of All Test Compounds with Biotin-Labeled DNA Sequences Using the Biosensor-SPR Method^a^

a *K*_coop_ (cooperativity index) = (*K*_A2_/*K*_A1_ × 4); *K*_coop_ = *K*_A2_/*K*_A1_ is 0.25 for the completely non-cooperative interaction. *K* = (√*K*_A2_ × *K*_A1_). NA = not applicable. All the results in the table were investigated in Tris-HCl buffer (50 mM Tris-HCl, 10 mM NaCl, 1 mM EDTA, 0.05% P20, pH 7.4) at 100 μL min^–1^ flow rate. The listed binding affinities are the average of two independent experiments carried out with two different sensor chips, and the values are reproducible within 10% experimental errors. 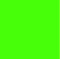 higher value; 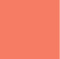 lower value.

We have made several intriguing findings regarding the BI/In–furan–furan derivatives DB1003 and DB1871. Replacing two thiophenes with two furan groups, DB1003 and DB1871, showed that these are weaker binders for the -AATT- binding site. DB1003 has an equilibrium binding constant, *K*_A_ of 0.2 × 10^7^ M^–1^, 100 times lower than the *K*_A_ value of DB2009 (Table 1). Although the indole analogue, DB1871, recovered some binding affinities for the -AATT- site (*K*_A_ = 0.7 × 10^7^ M^–1^) (Figure S4), it remained 20 times weaker than its indole analog DB1898. Based on SPR sensorgrams, the rate of association of DB1003 and DB1871 is very fast for the -AATT- binding site and the dissociation rate is also rapid (Figures 1 and S4). Interestingly, the sensorgrams reach the saturation plateau even at lower concentrations, which enabled us to fit 1:1 affinity plots for these weak binders. This result is consistent with other similar-sized compounds that bind to A-track sites, including -AATT-.^87,94^

The compounds containing BI/In–thiophene–furan, DB2868, and DB1897 possess moderately high binding affinities toward the -AATT- binding site. The SPR binding evaluation revealed that DB2868 and DB1897 exhibited affinities toward the -AATT- site with an equilibrium binding constant, *K*_A_ of 3.0 × 10^7^ and 6.7 × 10^7^ M^–1^, respectively (Table 1). Similarly, the BI/In–furan–thiophene compounds DB1992 and DB1896 demonstrated weaker binding affinities toward the same -AATT- binding site with a *K*_A_ value of 0.6 × 10^7^ M^–1^. Notably, SPR binding results indicate that the addition of the terminal thiophene ring in the DB1992 and DB1896 compounds has a significant impact. Curvature analysis (Figure S2) indicates that the addition of terminal thiophene partially restores the appropriate curvature (DB1992, 121^α^, and DB1896, 111^α^) (Figure S2) of the compounds and leads to moderate binders, DB1992 and DB1896, for the -AATT- binding site. These results emphasize the potential for enhancing binding affinities by manipulating the curvature of the compounds, as previously observed with DB818 (with a central thiophene) and DB293 (with a central furan).^87,93^

### SPR Binding Affinity Results for the -TTAA- Binding Site

Several previous studies have noted that DNA sequences with -AATT and -TTAA- binding sites have different physical and interaction properties. It is difficult for a monomeric heterocyclic diamidine to stack with the bases in the minor groove of the -TTAA- binding site. However, heterocyclic diamidines can bind to the minor groove by stacking with each other, and this requires favorable π-stacking of the diamidines. Although it has been challenging to achieve minor groove binding at -TTAA-, previous research discovered that DB293, a phenyl-furan-benzimidazole diamidine, binds as a cooperative dimer at -TTAA- but with no selectivity over monomer binding at the AATT sequence.^93^ After a thorough investigation, we have designed and synthesized a series of compounds (Scheme 1) with the goals of enhancing 5′-TTAA binding and reducing 5′-AATT binding.

In our initial design, we replaced the phenyl group with furan in DB293 and obtained a highly curved BI–furan–furan diamidine compound, DB1003 (Scheme 1a, and Figure S1). The SPR results reveal that DB1003 association and dissociation rates at the -TTAA- groove reached a steady-state plateau (Figure 1a). The plateau RU values indicate a 2:1 complex and are plotted using a two-site equation to obtain binding affinities (Figure 1b). The binding affinities and stoichiometries obtained from the SPR experiments showed that DB1003 binds to -TTAA- as a cooperative 2:1 complex with equilibrium binding constants *K*_A1_, 2.6 × 10^6^ M^–1^ and *K*_A2_, 75 × 10^6^ M^–1^. The cooperative binding results from the first DB1003 of the dimer indicate weak binding to the wider molar groove of -TTAA-. However, the second compound can stack with the first compound, and both compounds can interact with the walls of the -TTAA- minor groove, resulting in cooperative binding. The cooperativity factor (*K*_A2_/*K*_A1_ × 4) for the DB1003-TTAA complex shows that DB1003 has a relatively high cooperativity (*K*_coop(TTAA)_ = 115) at the -TTAA- binding site. The factor of 4 is included because two equivalent sites have *K*_A1_ = 4*K*_A2_ due to the lower entropy of the second site binding.

During the development of analogs of DB1003, changes have been made to the core five-member heterocyclic systems. SPR sensorgrams were compared by using the same compound concentration range for these compounds. In DB2009, both furan rings were replaced with two thiophene rings. Surprisingly, it has been observed that adding two thiophenes to DB2009 diminishes its stacking property at the -TTAA- minor groove. The result from SPR shows that DB2009 binds weakly (*K*_A_ = 1.6 × 10^6^ M^–1^) as a monomer (Figure 1 and Table 1) at the -TTAA- minor groove. This binding behavior is significantly different from that of its binding at the -AATT- site (Table 1).

The indole analogue of DB1003, DB1871, exhibits an average equilibrium binding constant (*K*) of 4.3 × 10^7^ M^–1^ for the -TTAA- site, which is 3 times higher than its parent compound, DB1003. DB1871 also forms a cooperative dimer with a similar cooperativity factor (*K*_coop(TTAA)_ = 112) as DB1003, indicating strong positive cooperativity for the -TTAA- minor groove (Table 1). The binding affinity results suggest that switching from benzimidazole to Indole increases the overall binding affinities toward DNA minor grooves. This phenomenon resulted in a similar sequence selectivity (*K*_TTAA_/*K*_AATT_ = 6) for DB1871. The indole analogue of DB2009, DB1898, also has increased binding affinity. However, it is interesting to note that similar to DB2009, DB1898 also binds as a weak monomer at the -TTAA- binding site with a *K*_A_ of 6.9 × 10^6^ M^–1^ (Table 1).

The following results suggest that the position of furans plays a critical role in the formation of strong cooperative dimers in -TTAA-, in the cases of DB1003 and DB1871. However, whether both furan rings are necessary for this effect is unclear. To investigate these phenomena, the central thiophenes in DB2009 and DB1898 were synthetically changed to furan in DB1992 and DB1896, respectively. Interestingly, DB1992 exhibits an average equilibrium binding affinity *K* of 14.8 × 10^6^ M^–1^ (*K*_A1_ of 1.7 × 10^6^ M^–1^ and *K*_A2_ of 1.3 × 10^8^ M^–1^) for -TTAA- that is 16 times higher than that of DB2009 and a similar binding affinity to DB1003 at the minor groove of the -TTAA- sequence (Figure 1). Even more strikingly, DB1992 stacks as a highly cooperative dimer (*K*_coop_ = 305) at the wider groove of -TTAA- (Table 1). The In–furan–thiophene analog, DB1896 represents a superior compound in this series. It exhibits the highest binding affinity for the -TTAA- sequence along with high cooperativity (*K*_coop(TTAA)_ = 438) and sequence selectivity (*K*_TTAA_/*K*_AATT_ = 10) (Table 1 and Figure S4). These unique properties of DB1992 and DB1896 highlight the influence of minor structural differences between heterocyclic diamidines in their ability to recognize DNA as dimers.

To better understand the importance of the central furan ring on the formation of stacked dimers, we synthesized two additional analogs, isomers DB2868 and DB1897 (Scheme 1). Based on the SPR sensorgrams and the binding plots, both compounds bind to the -TTAA- minor groove as monomers with relatively weak equilibrium binding constants (*K*_A_ = 1.0 × 10^6^ M^–1^ for DB2868 and *K*_A_ = 2.8 × 10^6^ M^–1^ for DB1897). This observation and others show that the presence of the central thiophene prevents the ligands from forming a stacked dimer at the minor groove of -TTAA-. MD results (see below) help understand this observation.

### SPR Binding Affinity Results for the -TATA- Binding Site

The minor groove width analysis reveals that despite the similarity in groove widths between the -TTAA- and -TATA- minor groove sites,^82^ the overall binding affinities of diamidine compounds (Figure 1) are lower for the -TATA- binding site than for the -TTAA- binding site (Table 1, and, Figure 1). For example, DB1003 and DB1871 exhibit almost eight times weaker binding for the -TATA-binding site than the -TTAA-binding site (Figures 1b, S4, and Table 1). A similar binding trend has been observed for BI/In–furan–thiophene compounds DB1992 and DB1896 (Table 1). However, BI/In–thiophene–furan compounds, DB2868 and DB1897, and BI/In–thiophene–thiophene (DB2009 and DB1898) bind weakly as monomers at the -TATA- minor groove, as observed for -TTAA- (Figure 1). The higher binding affinities of these stacked dimer compounds toward -TTAA- over -TATA- sites suggest that these ligands are highly sequence selective and require optimal groove width and five-membered heterocyclic aromatic systems to form a strong stacked dimer.

### Competition Electrospray Ionization Mass Spectrometry: Stoichiometry, Cooperativity, and Relative Affinity

The competition electrospray ionization mass spectrometry (ESI-MS) is a novel method that we have developed to provide comprehensive comparative information on the stoichiometry (directly from mass) and cooperativity when both 1:1 and 2:1 species are observed.^95,96^ This approach involves the simultaneous mixing of multiple DNA sequences with a small molecule to create a competitive binding environment, enabling a detailed comparison of DNA–ligand interactions. By maintaining the same free ligand concentration for all DNA sequences, the peak intensities of the unbound-DNA and DNA-small molecule complexes offer valuable insights into the preferred binding site(s). This technique can improve our understanding of molecular interactions and provide a critical test of ideas about the stoichiometry and selectivity.

**Figure 2:**
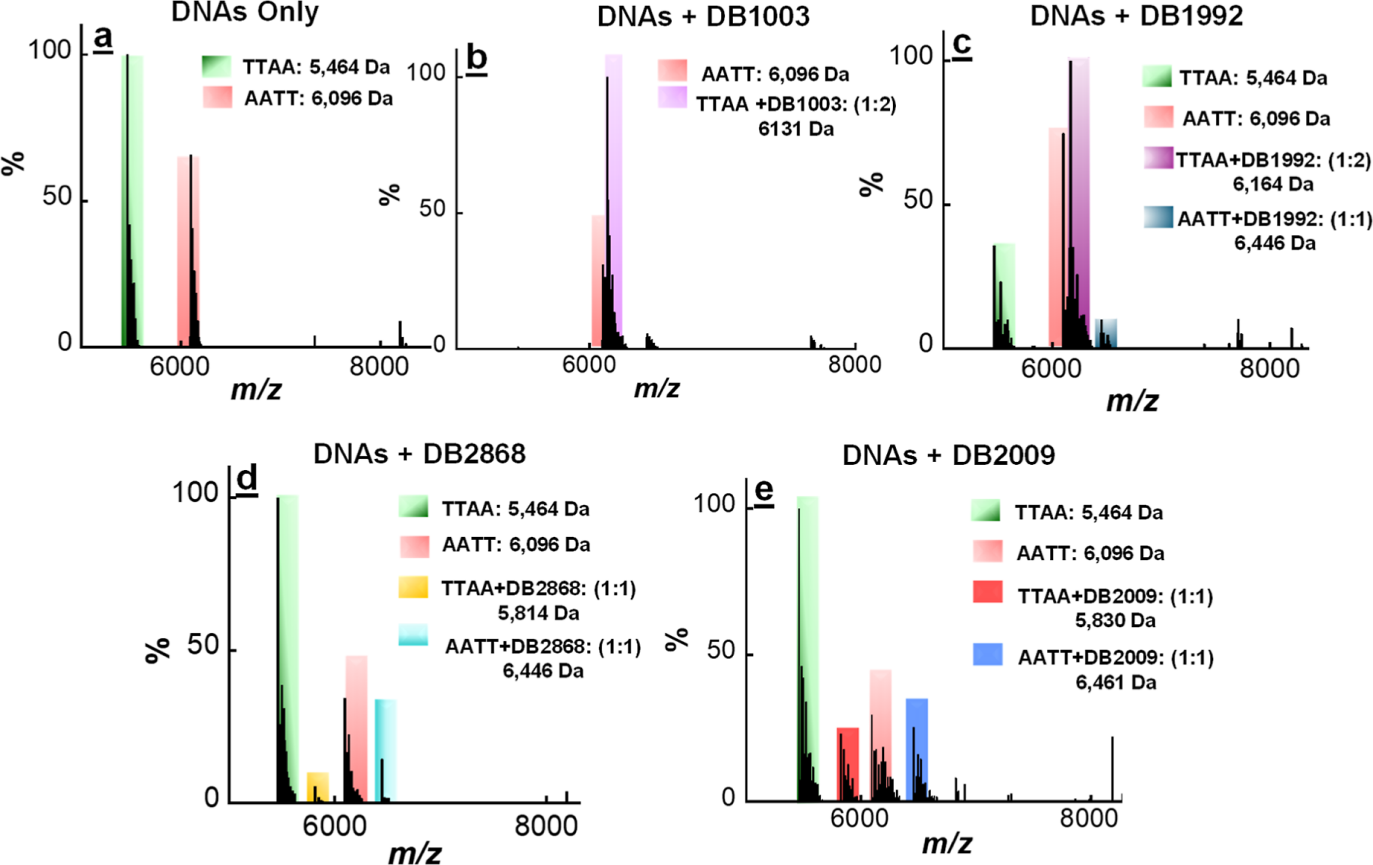
ESI-MS negative mode spectra of the competition binding of -AATT- and -TTAA- sequences (10 μM each) with 20 μM compound (DB1003, DB1992, DB2868, and DB2009, respectively) at a 2:1 ratio (compound to DNA) in buffer 100 mM ammonium acetate (NH_4_OAc) with 10% methanol (v/v), pH 6.8. (a) Mixture of -AATT- and -TTAA- DNA sequences; (b) -AATT- and -TTAA- DNA sequences with DB1003; (c) -AATT- and -TTAA- DNA sequences with DB1992; (d) -AATT- and -TTAA- DNA sequences with DB2868; and (e) -AATT- and -TTAA- DNA sequences with DB2009. The ESI-MS results are deconvoluted spectra, and molecular weights are shown at each peak. Full DNA sequences are in Scheme 1.

The spectrum in Figure 2a shows -AATT- and -TTAA- without any added ligand. Figure 2b illustrates DNA interactions with DB1003 at a [2:1] added ratio. The free -TTAA- sequence is no longer visible and shows that the -TTAA- has formed a 2:1, cooperative (DB1003)2–TTAA complex, as indicated by the tall peak at *m*/*z* 6132. There is no appearance of a -AATT- and ligand complex peak. The observed spectra clearly indicate the high specificity and affinity of DB1003 for the -TTAA- sequence and validate the results obtained from the SPR binding data.

With the -TTAA- sequence, DB1992 forms a high-intensity 2:1 complex (*m*/*z*, 6164), in agreement with the cooperative dimer formation in SPR. It is striking that there is only a very weak complex with -AATT–DB1992 (*m*/*z*, 6446) at this ratio, with most of the -AATT- left unbound, in direct agreement with SPR experiments. Mass spectra of DB2868 complexes show strong monomer binding (1:1) with the -AATT- sequence (*m*/*z*, 6446) (Figure 2d), in agreement with 1:1 binding observed in SPR. However, a very weak 1:1 complex with DB2868 is observed for -TTAA-, which is also in agreement with other biophysical methods.

The ESI-MS spectra of DB2009 and a mixture of -AATT- and -TTAA- sequences show that DB2009 makes a strong 1:1 complex (*m*/*z*, 6461) with a -AATT- binding site. This spectrum also shows a weak 1:1 complex with the -TTAA- sequence (*m*/*z*, 5830), which also supports monomer binding affinity from SPR (Table 1).

### Circular Dichroism

**Figure 3:**
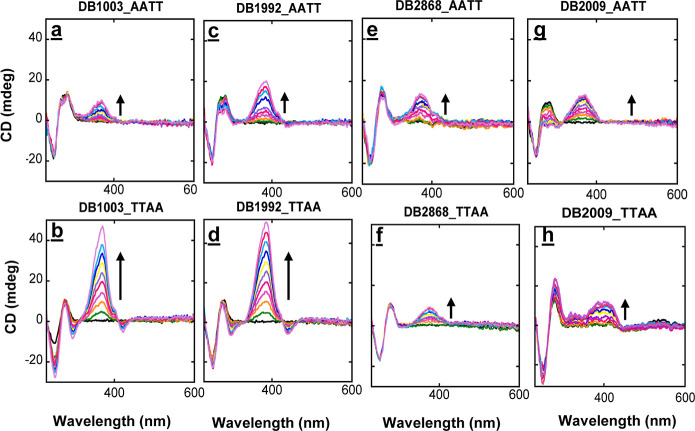
CD titration of -AATT- and -TTAA- containing DNA sequences with DB1003 (a,b), DB1992 (c,d), DB2868 (e,f), and DB2009 (g,h). The concentration of each duplex hairpin DNA is 5 μM, and each compound is added with 1 μM increments with a total of 10 increments. DB1003 (b) and DB1992 (d) with the -TTAA- sequence saturates at 2:1 compound to DNA ratio, confirming dimer formation for these compounds. However, DB2868 (f) and DB2009 (h) with the -TTAA- sequence saturates at a 1:1 compound-to-DNA ratio, confirming monomer complex formation for these compounds; (a,c,d,e) monomer formation of, respectively, DB1003, DB1992, DB2868, and DB2009 with the -AATT- sequence. The experimental buffer condition is TNE 100 (50 mM Tris-HCl, 100 mM NaCl, 1 mM EDTA, pH 7.4) at 25 °C. The arrows indicate the changes.

CD is a highly sensitive method for observing conformational changes in the helical structure of DNA and RNA and their complexes with small molecules.^97,98^ CD can also provide insights into small molecule binding modes with DNA through pattern recognition. CD spectra of the diamidine compounds in Scheme 1 when titrated with the -AATT- and -TTAA- sequences are shown in Figure 3. The addition of the compounds generates significant induced CD signals (ICD) between 300 and 450 nM, where the diamidines absorb and DNA signals do not interfere. This CD result indicates that the diamidine compounds used in this work (Scheme 1) bind as a monomer in the minor groove of the -AATT- sequence, as expected from the geometry of the compounds (Figures 3a,c,d,e; S5) and the SPR results. Moreover, incremental titration of ligands produces small and consistent spectral changes of standard *B*-form DNA at the CD region (230 to 290 nm), indicating only minor conformational changes in -AATT- upon complex formation. On the other hand, titrations of BI/In–furan–furan (DB1003 and DB1871) and BI/In–furan–thiophene (DB1992 and DB1896) with -TTAA- show very strong positive ICD changes in the compound absorption region above 300–450 nm, which reaches saturation near a 2:1 compound-to-DNA ratio (Figure 3b,d), in agreement with the SPR results. The presence of the isodichroic points around 430 nm wavelength for DB1003, DB1871, DB1992, and DB1896 (Figures 3b,d, and S5) indicates a two-state (DNA-bound and unbound) population. In summary, all compounds exhibit a minor groove binding mode for the -AATT- and -TTAA- DNA binding sites.

### X-ray Structure of the DB1992–TTAA Complex Structure

**Figure 4:**
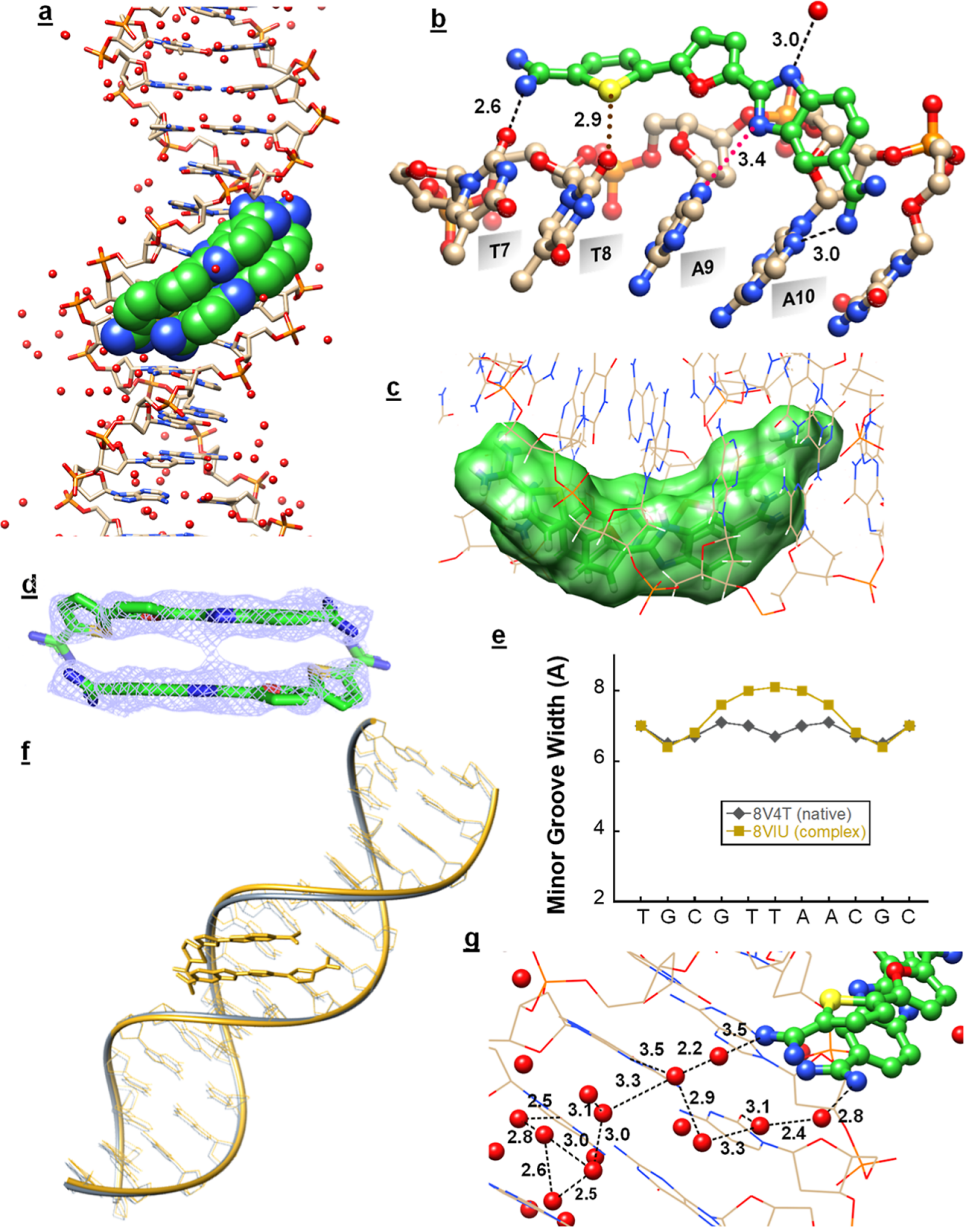
(a) Crystal structure of the antiparallel stacked DB1992 dimer with the -TTAA- sequence at 1.5 Å resolution (PDB ID 8VIU). The 16-mer -TTAA- containing oligomer is shown in stick form, DNA bases are represented in tan-white-blue-red (C–H–N–O) color, and stacked DB1992 molecules are shown in van der Waals representations with green-white-blue-red-yellow (C–H–N–O–S) color; (b) important interactions between different sections of the TTAA–DB1992 monomer with a symmetrical -TTAA- strand are shown; amidines from DB1992 form direct H-bonds with O2 of T7 and N3 of A10 (black lines), and the central BI–N has a long (3.4 Å) interaction with N3 of A9 (pink dotted line). The thiophene-S forms a chalcogen bond with O2 of T8 (2.9 Å, maroon dotted line), and the benzimidazole, BI–N, forms a direct H-bond with a water molecule (3.0 Å, black line); (c) surface representation of the antiparallel stacked dimer of DB1992 highlights the curvature of the dimer, which complements the curvature of the -TTAA- minor groove; (d) *2F*_o_ – *F*_c_ map for the antiparallel stacked DB1992 dimer in the minor groove of d(5′-GCTGCGTTAACGCAGC-3′)2; (e) comparison of minor groove widths in the crystal structures of the native TTAA-containing duplex DNA sequence (PDB ID, 8V4T) and the -TTAA- containing sequence with DB1992 dimeric complex (PDB ID 8VIU); (f) overlay of the structure model of the native -TTAA- containing sequence (PDB ID, 8V4T, gray color) and -TTAA- sequence with the DB1992 dimeric complex (PDB ID 8VIU, gold color); (g) extended water network interaction occurring at each end of the DB1992 dimer involves DNA bases, as well as the DNA backbone.

The high-resolution crystal structure of the DB1992-d(5′-GCTGCGTTAACGCAGC-3′)2 (PDB ID: 8VIU, 1.5 Å) complex reveals that DB1992 binds to the central -TTAA- DNA site as a 2:1 minor groove complex (Figure 4a–d), having exact (crystallographic) 2-fold symmetry (Table S1) such that the asymmetric unit is a single DNA strand and one DB1992 molecule. The structure shows that there is a significant widening of the -TTAA- minor groove width compared to the native DNA X-ray structure (PDB ID 8V4T) (Figure 4e,f) by > 1.5 Å. The crystallization conditions for the native structure were similar to those for the complex.

In the ligand-bound structure, the two DB1992 molecules are stacked together in an antiparallel arrangement that slides deeply into the groove and closely matches the groove curvature (Figure 4a,c). The two furan oxygen and two thiophene sulfur atoms point into the groove, as expected from the compound curvature (Figure 4c). The two compounds also stack against opposite walls of an expanded minor groove. Also, the compound makes additional contacts to the edges of DNA bases that face into the minor groove.

Because of the 2-fold symmetry of the structure, the two compounds make similar contacts with the DNA bases. The terminal thiophene amidine groups form an H-bond to the O2 of T7 in the minor groove (2.6 Å, Figure 4b).The benzimidazole amidine at the opposite end of the molecules forms an H-bond to N3 of A10 (3.0 Å, Figure 4b). The thiophene S forms a chalcogen bond with the minor groove O2 of T8 (2.9 Å; Figure 4b). The BI–N that points into the groove and has a long interaction with N3 of A9 (3.4 Å). However, MD simulation of the TTAA–DB1992 dimeric complex reveals that (see below) BI–Ns form a stable H-bond interaction with DNA minor groove base with ∼3.0 Å bond distances in the 1 μs simulation run. The BI–N that points out of the groove forms a strong hydrogen bond to a water molecule (3.0 Å). This gives six strong contacts between the stacked dimer and the -TTAA- minor groove.

Additionally, there is an irregular cluster of water molecules at each end of the dimer, some of which mediate contacts between amidinium groups and DNA base edges (Figure 4g). Others also interact with O4′ deoxyribose ring oxygen atoms. Part of the cluster extends along the groove for another two base pairs, making extensive DNA contacts. Finally, the entire dimer complex has favorable interactions with the walls of the minor groove at the -TTAA- site.

DB1992 binds more weakly to the DNA minor grooves in the -AATT- sequence, whose narrower groove is more appropriate for monomers. This was illustrated in the first crystal structure of a minor groove complex in -AATT- with bound netropsin (and in many subsequent AATT minor groove complexes).^99^ Netropsin binds in a 1:1 complex with favorable H-bond contacts to the A and T base edges. This complex also had excellent stacking with the walls of the minor groove in -AATT-.

### MD Simulations

#### DB1992–TTAA MD

To gain a better understanding of the binding structure and dynamics of the BI–furan–thiophene compound, DB1992, at the -TTAA- binding site, a 1 μs MD simulation using d(5′-GCTGCGTTAACGCAGC-3′)2 DNA was carried out. This approach provides a test of the findings from the X-ray structure of the (DB1992)2–TTAA complex. Moreover, it can unambiguously identify additional structural models and dynamic transitions compatible with the biophysical results.

**Figure 5:**
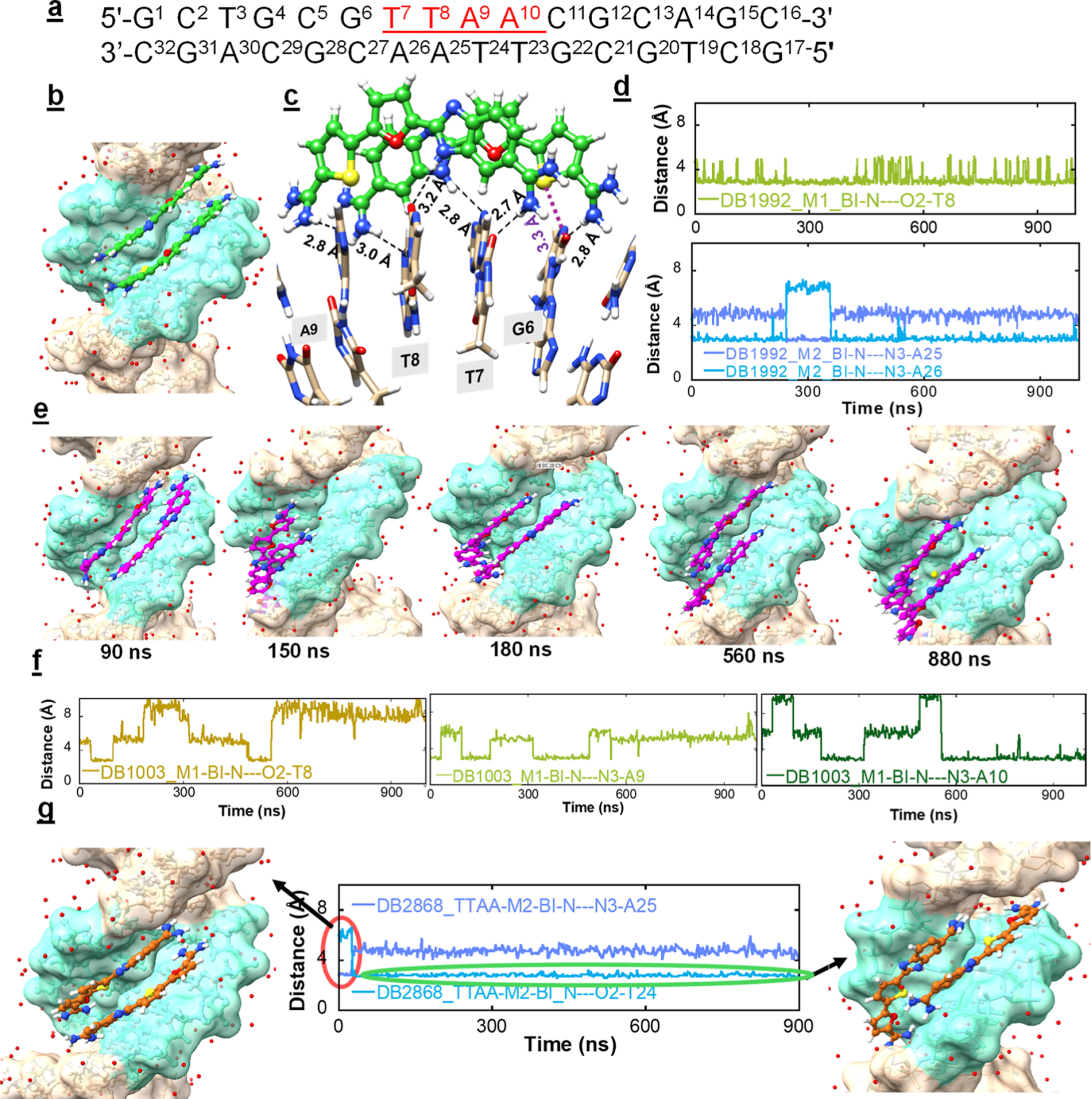
(a) DNA sequence used for MD analysis; the highlighted bases in red indicate the binding site of the ligands; (b) a surface model viewed into the minor groove of the -TTAA- binding site with bound stacked DB1992 dimer; the DNA bases at the -TTAA- site are represented in aquamarine, and the rest of the sequence is colored tan; the stacked DB1992 dimer is colored green-white-red-blue-yellow (C–H–O–N–S); The significant interactions between different sections of the DB1992-dimer and DNA complex are illustrated in (c); two stacked DB1992 (DB1992-M1 and DB1992-M2) form six direct hydrogen bonds (black dashed lines) with DNA bases; the direct interactions are (i) terminal amidines with N3 of A9 (DB1992-M1) and N3 of A26 (DB1992-M2), (ii) central BI–N with O2 of T8 (DB1992-M1), and N3 of A25 (DB1992-M2), (iii) other terminal amidines with O2 of T7 (DB1992-M1) and O2 of C27 (DB1992-M2), and (iv) thiophene-S forms a chalcogen bond with O2 of T8 (2.9 Å, purple dotted line); (d) distance plots between BI–N (DB1992-M1)-O2 of T8 (top) and between BI–N (DB1992-M2) with N 3s of A25 and A26; (e) surface model viewed into the minor groove of the -TTAA- binding site with the bound stacked DB1003 dimer; coloring is as above; the dynamic behavior of the stacked DB1003 dimer at the -TTAA- minor groove dimer is illustrated here; (f) distance plots between BI–N (DB1003-M1)-O2 of T8 (left) between BI–N (DB1003-M1)-N3 of A9 (middle) and between BI–N (DB1003-M1) with N3 of A10; (g) surface representation viewed into the minor groove of the -TTAA- binding site with the bound DB2868 dimer; the DNA bases at the -TTAA- site are represented in an aquamarine color scheme, and the rest of the sequence is represented in tan; the DB2868 dimer is in a brown-white-blue-yellow (C–H–N–S) color scheme; the distance plot between BI–N (DB2868-M2)-N3 of A25 and O2 of T24 (middle); the red circle represents the initial 32 ns of simulations where two DB2868 form the original stacked–docked complex. The green circle represents the remainder of the simulation. In this final region of the simulation, the two molecules of the DB2868 dimer slide away from each other and are much less overlapped. In distance plots, the time interval for each frame in the plot is 20 ps of a 1 μs trajectory (total 50,000 frames).

The detailed simulation procedures are described in the Materials and Methods section. Through the MD trajectory, structures were collected every 20 ns to determine which major features of the DB1992–DNA complex contribute most to its excellent stability (Table 1) and high cooperativity (*K*_coop_ = 376). The MD simulations of the (DB1992)2–TTAA complex showed that the antiparallel stacked dimer of DB1992 at the -TTAA- minor groove is highly stable but with some dynamic features. Both compounds are tightly bound at the -TTAA- groove walls and produce optimum H-bonding distances with DNA bases. There are six optimum H-bonds in the complex. Four amidine–N–H groups of the dimer form strong H-bonds with N3 of A or the O2 of T (Figure 5b,c) with an average of 2.8 Å in H-bond distances. As in the X-ray crystal structure, two thiophene-sulfur atoms form strong chalcogen bonds with O2 of Ts. The simulation results for DB1992–TTAA reveal a high level of cooperative stacking between the two ligand molecules. For most of the simulation, the distance between the two BI unprotonated N atoms was approximately 3.5 Å (Figure S6), indicating that there is excellent antiparallel stacking between DB1992 compounds. The amidine groups are highly dynamic and frequently rotate 180°; however, rotation is rapid, and the amidines always form strong H-bonds with the DNA bases. The other two stable H-bonds are from the two BI–H bonds of DB1992 (Figure 5c). For most of the time, both BI–H are directly linked to O2 of T (M1 of DB1992) and N3 of A25/A26 (M2 of DB1992) with an average of 3.0 Å in H-bond distances.

The amidines also form numerous highly dynamic extended hydrogen bonds to water molecules that move in and out of the minor groove. These water molecules also frequently form hydrogen bonds with A·T base pairs at the floor of the minor groove, helping to link the compound to a specific binding site in the groove and stabilize the complex. Similar results have been seen with other minor groove binders.^4^

### MD Simulation of the TTAA-DB1003-Dimer

#### Both Furan Oxygen Atoms Oriented toward the Minor Groove

We also crystallized the DB1003 and -TTAA- DNA sequences. However, we have yet to obtain a high-resolution X-ray structure for this complex. A low-resolution structure of the complex (DB1003)2-d(5′-GCTGCGTTAACGCAGC-3′)2 shows that two DB1003 are stacked as an antiparallel dimer at the minor groove of the -TTAA- region. Two antiparallel DB1003 molecules were initially docked at the same location on the d(5′-GCTGCGTTAACGCAGC-3′)2 duplex sequence, as observed for two DB1992 molecules in the X-ray structure. These molecules are stacked as dimers at the same position as in the X-ray structure for the (DB1992)2–TTAA complex. In this docked structure, both furan groups of each compound are oriented in the same direction as the BI–NH group. The 1 μs MD simulation revealed that the two bound DB1003 molecules remain stacked and close to the -TTAA- site and form strong H-bonds at the -TTAA- minor groove through most of the MD simulation (Figure 5e).

Interestingly, in the (DB1003)2–TTAA simulation, it has been observed that the two DB1003 molecules slide back and forth over each other more dynamically than is observed in the DB1992 complex. From 860 to 870 ns, one end of one molecule swings slightly away from the floor of the minor groove, and an interfacial water is captured between the amidine that has moved away from DNA and the floor of the minor groove (Figure 5e,f). The swing-out also moves the BI–NH away from the DNA, and subsequently, this water jumps to the BI–NH to link it to the minor groove.

Moreover, the amidine then captures another water so that both amidines and BI–NHs are linked to DNA through interfacial waters. The word jump is used here to describe the water movement because no intermediate non-H-bonded water frames were observed. Both bound waters are very dynamic and interchange rapidly with bulk water. The amidine-water ratio varies rapidly from interfacial to terminal. This type of dynamic water enhances the binding entropy.

At about 890 ns, one of the amidines again forms a direct H-bond with DNA, but the BI–NH holds water, and the other amidine moves away from DNA in a highly dynamic manner. The system is similar at 900 ns trajectories, although at this point, the amidine only has terminal, not interfacial, associated water. The binding Gibbs free energy loss caused by highly dynamic sliding and interfacial water-mediated H-bond formation leads DB1003 to be a weaker ligand for the -TTAA- minor groove than DB1992. The dynamic nature of the complex also helps to explain the low-resolution X-ray structure.

#### One Furan Oxygen Pointing toward the Minor Groove, and the Other Oxygen Pointing out of the Minor Groove

In this MD simulation, two DB1003 molecules were docked antiparallel, but the central furan oxygen was pointed into the minor groove, while the terminal furan oxygen was pointed out of the minor groove. This structure differs from that of the DB1992 complex but cannot be rejected because of the low-resolution X-ray structure with DB1003. The two stacked compounds form five H-bonds (2.7–3.1 Å) in this arrangement, with one furan-amidine being about 5 Å from any acceptor group on the floor of the minor groove. This amidine captures a water molecule and completes the H-bonding between the compounds and DNA in the complex. This arrangement with small dynamic movements is maintained until for 150 ns of MD simulation time. However, around 180 ns, both the furan and the amidines dynamically move out from the floor of the minor groove (Figure S7).

By 190 ns, the two molecules slide along one another, reducing the stacking surface (Figure S7). One molecule also starts to slide out of the minor groove. In this arrangement, the compounds thus slide along each other and move the unprotonated BI–N apart by 6–7 Å, with one molecule dynamically moving away from the minor groove stacking arrangement (Figure S6). By 300 ns, the stacking overlap surface of the two molecules is reduced by over 50%. This trend is maintained until 400 ns. The loss of several strong interactions between the DB1003 dimer and DNA means that the DB1003 structures are not effectively bound in a minor groove complex. At least one or two highly dynamic water molecules were captured between each DB1003 and the minor groove surface of DNA (Figure S7). After 300 ns, one BI dynamically complexes a water molecule in order to interact with DNA. At the same time, two amidines have acquired interfacial waters that link to DNA bases. These weak interactions hold the dimer in the area of the minor groove, but this complex is considerably weaker than the BI/In–furan–furan dimer complex, where both furans face the same directions with BI–N–H. The complex is thus not considered a preferred orientation of the compounds based on the loss of overlap and direct H-bonds from the compounds to the minor groove bases in the MD analysis.

### MD Simulation of the TTAA-DB2868-Dimer

To better understand why compounds with a BI–thiophene–furan, such as DB2868, bind weakly as dimer complexes to the -TTAA- minor groove and have no positive cooperativity binding, we also investigated the DB2868 complex by an MD simulation.

The two DB2868 molecules were docked at the TTAA minor groove in an antiparallel manner, similar to what was observed in the crystal structure of the stacked DB1992 dimer with the -TTAA- sequence (PDB ID 8VIU). During the MD analysis, at 32 ns (2000–3000 frames), a significant separation was observed between the two molecules. The MD trajectories plot demonstrates (Figure 5g) that after 32 ns of simulation, the two molecules slid away from each other, causing the dimer to have a reduced overlap. Surprisingly, for the rest of the 1 μs simulation time, the two molecules stayed in a noncooperative dimer configuration. At this point, the BI unprotonated Ns were now 5–6 Å apart, which is an unsuitable arrangement for a three-heterocyclic ring compound to form a strongly stacked dimer (Figure S6). The thiophene and furan rings were unstacked with respect to the other elements of the dimer. This essentially unstacked arrangement was maintained with only small dynamic changes over the remainder of the trajectory. The two BI six-member rings were stacked over each other, and the BI-imidazoles are stacked primarily over the opposite molecule, BI-amidine. The -TTAA- minor groove in this complex is wide enough to accommodate the dimer, but the stacking was minimal (Figure 5g). This arrangement helps explain the poor binding of DB2868 to -TTAA- compared to its isomer, DB1992. In all cases, compounds with a central thiophene yield poorly stacked complexes. Note, for example, the weak binding of DB1897 and DB1898 in addition to that of DB2868 (Table 1).

## Discussion

The DNA minor groove complexes of the ligands in Scheme 1 and Table 1 and their interactions with different DNA sequences illustrate several important features of minor groove compounds that have not previously been observed in a single set of agents and complexes: (i) DNA binding to AT sequences with significantly different minor groove widths has quite different interactions with the compound set. Several studies have illustrated that A-tract sequences, such as -AAAA- or -AATT-, have narrow minor grooves with strongly coordinated water molecules (i.e., a spin of hydration) in the groove, while wider minor grooves are observed in sequences such as -TTAA- and -TATA- with less tendency to form coherent patterns of structured water molecules.^47,84^ As a result, the minor grooves at the -TTAA- sequences are more readily expanded to accommodate a dimer complex; (ii) there is a critical interplay between the ligand structures and DNA sequences; for example, DB2009 with two thiophenes exhibits a relatively weak monomer binding (*K*_A_ = 0.1 × 10^7^ M^–1^) with -TTAA-. In contrast, it shows a significantly higher monomer binding constant of 24 × 10^7^ M^–1^ with -AATT- while DB1003 with two furans has an average cooperative binding constant of 1.3 × 10^7^ M^–1^ with -TTAA- but a lower affinity for AATT of 0.2 × 10^7^ M^–1^; (iii) in addition to complementary molecular curvature, which is very important for minor groove binding, aromatic molecular thickness perpendicular to the aromatic ring and its influence on stacking energetics in dimer complexes must be considered with this compound set-even small differences in covalent molecular thickness can have significant effects when compounds bind to the minor groove as stacked dimers; (iv) the position and type of groups in two isomers can have significantly different effects on affinity; for example, as shown in Table 1, X–furan–thiophene and X–thiophene–furan, where X can be benzimidazole or indole, bind with quite different affinities to -AATT- and -TTAA- containing sequences. These results clearly illustrate how small differences in compound structure interact with different AT DNA sequences to generate a range of affinities. To summarize, previous diamidine results defined the importance of molecular curvature on minor groove complexes (Figure S2), but the importance of small differences in molecular aromatic thickness is new and can be clearly seen in comparing dimer complexes of compounds with central thiophene or furan systems.

In our design, search for synthetic compounds that could efficiently and selectively bind to the wider grooves of -TTAA- and -TATA- sequences; compounds with a benzimidazole or indole linked with two furans form stacked dimers to effectively bind to -TTAA- over -AATT-. This observation raises the question of whether the difuran module has especially favorable properties for stacking and binding to -TTAA- or could other five-member heterocycles, such as thiophene, also form stacked dimers with effective binding to -TTAA- sequences? To answer this question, all possible combinations of furan and thiophene diamidines with either benzimidazole or indole groups were synthesized (Scheme 1). The eight compounds in Scheme 1 were designed to probe the types of heterocyclic benzimidazole/indole diamidines that can bind strongly as stacked, antiparallel dimers at the -TTAA- and -TATA- minor grooves. Our design concept focuses on heterocyclic diamidines that offer excellent solubility, cell uptake, and feasible synthesis (for example, the synthesis scheme of DB2868 in Supporting Information).^2,17,18,34,36,65,67,89,90^

As expected from their shape, chemical groups, and charge, CD studies show that the new compounds (Scheme 1a) bind to the minor grooves of -AATT-, -TTAA- and -TATA- sequences (Scheme 1b). The results of biosensor-SPR experiments with -AATT-, -TTAA- and -TATA- DNA sequences revealed that all compounds in Scheme 1a have moderate to strong binding affinities that vary with the different sequence-dependent minor groove widths (Scheme 1c).^82^ The control compound, DB1003, and its indole analogue, DB1871, have been found to bind strongly to -TTAA- sites as dimers. Both compounds interact with the reverse sequence -AATT-, significantly more weakly than -TTAA- and as monomers. These initial results and the quite different binding results to -AATT- and -TTAA- sequences show that designing compounds with specificity for -TTAA- over -AATT- containing sequences is possible. The majority of dications do not form stacked dimer complexes with DNA minor grooves due to charge repulsion. However, DB1003 defies this trend by forming a strong positive, cooperative stacked dimer at the -TTAA- minor groove (Table 1 and Figure 1). The 2:1 binding ratio of DB1003 to the -TTAA- sequence is further evidenced by the notably strong induced CD (ICD) signal within the 300–400 nm wavelength range, as shown in Figure 3. The arrangement of a stacked dimer of DB1003 within the -TTAA- minor groove demonstrates that the repulsion of the dicationic charge in the stacked DB1003 complex with TTAA requires significantly less Gibbs energy (Δ*G*°) than the energy gained from the amidine interactions in the complex. The negatively charged backbone of DNA effectively shields the four positive charges of the stacked dimer, allowing it to form within the TTAA minor groove.

As has been noted previously with DB818,^87,88^ changing the furan to thiophene results in the ligand having lesser curvature, and as observed from SPR results with AATT, DB2009, and DB1898 bind with a fast association constant and a slow dissociation rate. These results imply that the presence of the BI–thiophene-heterocyclic moiety gives the ligand an optimum curvature to fit the minor groove of -AATT- (Figure S2). On the contrary, the affinities for -TTAA- were significantly reduced, but in addition, DB2009 and DB1898 failed to form an optimum stacked dimer in the -TTAA- minor groove. Significant negative cooperativity exists in the interaction with the -TTAA- site due to unfavorable stacking effects from the two sulfur atoms in the thiophene rings. This effect is seen in all compounds of Scheme 1 that have a central thiophene. It was observed in MD simulation that these compounds can be docked in a stacked conformation but quickly slide apart (Figure 5g), reducing the DNA affinity for the central thiophene dimers. In stacked dimers of compounds with terminal thiophene, such as DB1992, the thiophenes do not come in contact and do not reduce the affinity of the compounds for the -TTAA- site. As seen in Table 1, the dimer binding of DB1003 (benzimidazole–furan–furan) and DB1992 (benzimidazole–furan–thiophene) are similar. However, the indole derivative of DB1992, DB1896, binds slightly more strongly than the benzimidazole isomers (Table 1).

The crystal structure data show that DB1992 forms an antiparallel dimer with strong direct hydrogen bonds to DNA bases in the -TTAA- site (Figure 4a). This is the first dimer crystal structure of a DNA complex with a diamidine minor groove binding compound and the first crystal structure at a -TTAA- site. The two ligands in the crystal fit well between opposite walls of the -TTAA-minor groove and can be twisted to match the groove curvature. The two BI–N–Hs form strong H-bonds (∼3.2 Å) with either the O2 of T or N3 of A (Figure 4b) that project into the minor groove. The two S atoms from thiophene also give additional stability by forming chalcogen bonds with O2 of Ts. Due to the optimum curvature and the size of DB1992 (Figure S2), it is able to minimize the dicationic repulsion and generate a very stable stacked dimer, which has also been observed from the MD simulation (Figure 5b–d).

MD analysis of two DB1992 molecules bound to a -TTAA- site reveals some very interesting features of the complex that are difficult to obtain solely from the experimental analysis. As seen in the X-ray structure, MD simulations also show strong and long-lasting DB1992–TTAA H-bond interactions (Figure 5d). Unlike DB1003 (Figure 5e,f), DB1992 binding results in a highly stable stacked dimer with very little sliding characteristic at the minor groove of the -TTAA- sequence. The enhanced stability of two DB1992 molecules at the -TTAA- site undoubtedly contributed to the successful crystallization and high-quality crystal structure of the DB1992–TTAA- complex. These compounds were specifically engineered to interact with the -TTAA- groove in this manner, forming an antiparallel stacked dimer complex. The -BI/In–furan- group evidently serves as a crucial binding motif, forming a stacked antiparallel dimer with a substantial affinity for -TTAA-. On the contrary, in the analysis of the MD results for DB1003-TTAA, it was observed that despite the formation of a robust cooperative dimer by DB1003, the highly curved ligand dimer structure (Figure S2) led to the positive charges of the diamidines being in close proximity. As a result, the minor groove binding interaction of DB1003 exhibited a dynamic hopping feature (Figure 5e). All of these findings shed light on the significance of positioning the thiophene and furan rings. The observation that the central thiophene ring plays a crucial role in preventing the formation of a stacked dimer, as evidenced by MD simulations, is a significant new finding from these studies.

Central thiophenes are excellent for adjusting the curvature of minor groove binders to fit well into the minor grooves of AATT sequences. However, they are not well suited for forming diamidine dimers due to the size of the sulfur atom in the central thiophene, which does not favor stacking interactions. This can be seen by comparing compounds with central thiophenes in Table 1. They are excellent groups for binding -AATT- (curvature) but are very poor at stacking at -TTAA- (aromatic asymmetry thickness).

## Conclusions

We report the differential binding of the eight synthetic diamidines, shown in Figure 1, to hairpin DNA molecules with either an -AATT- or an -TTAA- binding site. This is part of a larger program to design, prepare, and test minor groove binding compounds for the recognition of specific DNA sequences.^33,35,37,39^ The binding mode, affinities, and kinetics of the compounds to DNA were evaluated by using SPR, ESI-MS, and CD methods. An X-ray structure of a stacked dimer bound to -TTAA- is provided for DB1992, a benzimidazole–furan–thiophene. This structure illustrates how the -TTAA- groove can be widened to accommodate a stacked dimer. The dynamic flexibility of the -TTAA- site allows it to open for dimer formation.

The ability of noncovalent minor groove binders to interrogate particular DNA sequences is central to their potential role as therapeutic agents in a wide range of diseases.^2,3,12,15,17,18,34,44,45,51,53,59,65,66,100−106^ A minor groove binder that has been assessed in human clinical trials is the diamidine compound furamidine (DB75) and its orally active analog pafuramidine. They have been evaluated against human African trypanosomiasis^43−45^ and have shown high potency but also liver and kidney toxicity in some volunteers. To date, they have not progressed beyond phase III clinical trial evaluation. The symmetric bis-benzimidazole compound ridinilazole^100,101^ (SMT19969) has been evaluated in a phase III trial for human *Clostridioides difficile* hospital-based infections.^102,103^ This MGB has a narrow spectrum of activity and is more effective at preventing recurrence of disease than the best current treatment (vancomycin). Even though its antibacterial activity is not superior to that of vancomycin, it was concluded that ridinilazole is a well-tolerated and safe drug. This outcome demonstrates that human toxicity is not an intrinsic property of noncovalent MGBs but appears to be compound-specific.

Furamidine and ridinilazole are both structurally symmetric minor groove binders and both have high affinity for AATT sites in duplex DNA, as assessed by biophysical and crystallographic methods.^104,105^ This sequence preference is associated with selectivity for regulating the expression of organism-critical genes that have AATT sites in their promoters, blocking the binding of AATT-selective transcription factors.^104^ We suggest that the development of minor groove binders such as compound DB1992, with systematically engineered sequence preferences, will enable the effective targeting of appropriate sequences in key genes for a range of bacterial and parasitic diseases. This has been successfully demonstrated with polyamides, targeting a range of transcription factor sites and sequences (see, for example, 106). It remains to be demonstrated that the benzimidazole–furan–thiophene group is an effective pharmacophore. However, it is well-established that its individual component groups possess drug-like properties (as shown by, for example, the benzimidazole and furan groups in ridinilazole and furamidine). So one can be optimistic about the potential therapeutic utility of future compounds derived from DB1992.

## Materials and Methods

### Chemistry

The syntheses for previously reported diamidines are provided in the references cited: DB1003, DB1871, DB1992, DB1896, DB2009, DB1898, and DB1897.^78^

The synthesis of unreported DB2868 has been described below.

#### 2-(5-(5-Cyanofuran-2-yl)thiophen-2-yl)-1*H*-benzo[*d*]imidazole-6-carbonitrile (**2**)

Sodium metabisulfite (1.14 g, 6 mmol) was added to a solution of 3,4-diaminobenzonitrile (0.40 g, 3 mmol) and 5-(5-formylthiophen-2-yl)furan-2-carbonitrile **1** (0.60 g, 3 mmol) in DMSO (10 mL), and the mixture was heated at 150 °C for 30 min. Then, the reaction mixture was poured into water, filtered, and dried. Purification was by crystallization from DMF.

Yellow solid (0.73 g, 78%), mp > 300 °C; ^1^HNMR (600 MHz, DMSO-*d*_6_): δ 8.08 (br s, 1H), 7.89 (d, *J* = 3.6 Hz, 1H), 7.70 (m, 3H), 7.58 (m, 1H), 7.18 (d, *J* = 3.6 Hz, 1H). Anal. Calcd for C_17_H_8_N_4_OS: C, 64.55; H, 2.55; N, 17.71. Found: C, 64.48; H, 2.59; N, 17.57.

#### 2-(5-(5-Carbamimidoylfuran-2-yl)thiophen-2-yl)-1*H*-benzo[*d*]imidazole-6-carboximidamide (**3**) (DB 2868)

A mixture of hydroxylamine hydrochloride (1.28 g, 18.4 mmol) in anhydrous DMSO (10 mL) was cooled to 5 °C under nitrogen, and potassium *t*-butoxide (2.06 g, 18.4 mmol) was added in portions. The mixture was stirred for 30 min and added to dinitrile **2** (0.57 g, 1.84 mmol) in DMSO (5 mL). The reaction mixture was stirred overnight at RT and poured slowly onto ice water (100 mL). The precipitate was filtered, washed with water, and dried. To a solution of the formed amidoxime in glacial acetic acid (15 mL), acetic anhydride (0.5 mL, 5.52 mmol) was slowly added. After stirring overnight, TLC indicated complete acylation of the starting material, the solvent was removed under reduced pressure, and 10% palladium on carbon (0.3 g) was added to the acetoxy derivative in 40 mL glacial acetic acid/ethanol (1:3). The mixture was shaken in a Parr hydrogenation apparatus at 50 psi for 12 h at RT. The mixture was filtered through Celite, and the filter pad was washed with water. The filtrate was evaporated under reduced pressure, and the precipitate was collected and washed with ether. The obtained precipitate was neutralized with NaOH solution, filtered, and dried. The free base was suspended in ethanolic HCl, stirred for 24 h, and concentrated; diethyl ether was added, and the formed HCl salt was filtered and dried under a vacuum at 100 °C for 12 h.

Yellow solid (0.51 g, 56%), mp > 300 °C. ^1^HNMR (600 MHz, DMSO-*d*_6_): δ 9.49 (s, 2H), 9.37 (s, 2H), 9.13 (s, 2H), 9.09 (s, 2H), 8.10 (m, 2H), 7.90 (m, 2H), 7.68 (m, 2H), 7.24 (br s, 1H). Anal. Calcd for C_17_H_14_N_6_OS·3HCl·2H_2_O: C, 41.18; H, 4.26; N, 16.95. Found: C, 41.11; H, 4.21; N, 16.88.

### Biophysical Experimental

The details for materials and sample preparation for biophysical experiments and experimental procedures of biosensor-SPR, CD, competition ESI-MS, and molecular curvature determination are described in Supporting Information.

### DNA Crystallization

16-mer DNA oligomer, d(5′-GCTGCGTTAACGCAGC-3′)2 was purchased from Integrated DNA technologies. The DNA was dissolved in 7.5 mM HEPES, pH 6.6 buffer, and annealed by heating to 95 °C and slowly cooling to RT. DNA stocks were diluted to 1 mM concentration for crystallization with 7.5 mM HEPES, pH 6.6 buffer, and aqueous stock solution of diamidine compounds to a final concentration of 3.5 mM. The ligand solutions were gently warmed to 42 °C for 1 h before crystallization to aid ligand incorporation. Crystals were grown by mixing prepared duplex 1:1 with well solution containing 10 mM HEPES, pH 8.6, 600 mM CaCl_2_, and 34–46% PEG 400 (ligand-bound typically required higher concentrations). Crystals grew over 1–3 days and were looped and flash-frozen directly for X-ray data collection.^28,41^

### X-ray Data Collection and Processing

X-ray diffraction data was obtained at the Brookhaven National Laboratory on the 17-ID-2 beamline. Data sets were collected at 100 K, and the data were initially auto processed with XDS. Data reduction was carried out using Aimless in the CCP4i2 suite. Molecular replacement was run with PHASER-MR in the Phenix suite under maximum-likelihood search procedures, and models were refined through iterative cycles of refinement in phenix.refine. For ligand fitting, a clear region of extra density could be observed in the data, but the orientation of the small molecule proved to be challenging upon initial assessment. The best-fit model was obtained by fitting 4 potential orientations and using minimized *R*_free_ values to guide fitting. Information regarding specific collection parameters for each data set can be found in the crystallographic information Table S1.^28,41^

### MD Simulations

Structure optimization of DB1992, DB1003, and DB2868 was performed by using DFT/B3LYP theory with the 6-31+G*(d,p) basis set in Gaussian 13 (Gaussian, Inc., 2009, Wallingford, CT) with Gauss-view 5.09.^107^ Partial charges were derived using the RESP fitting method (restrained electrostatic potential),^108,109^ and the AMBER 16 (Assisted Model Building with Energy Refinement) software suite was used to perform MD simulations^110^ The initial coordinate of canonical *B*-form d(5′-GCTGCGTTAACGCAGC-3′)2 DNA was taken from the X-ray structure (PDB ID, 8V4T). The ANTECHAMBER tools^94^ were used to create *LEaP* input topology files for the DB1992, DB1003, and DB2868. Specific atom types assigned for DB1992, DB1003, and DB2868 molecules were adapted from the *ff99* force field. Most of the force field parameters for these molecules were derived from the existing set of bonds, angles, and dihedrals for similar atom types in *parm99* and *GAFF* force fields.^111^ Some dihedral angle parameters were obtained from previously reported parametrized data.^112−114^ The molecular structures with specific atom types used for the DB1992, DB1003, and DB2868 molecules are shown in Figures S8–S10, respectively. The parameters of these three in the.frcmod file are listed in Tables S2–S4.

The AutoDock Vina^115^ program was used to dock two DB1992 stacked dimers, with the same bases in the minor groove of DNA observed from the X-ray structure of the TTAA-(DB1992)2 complex (PDB ID 8VIU). For DB1003 and DB2868, similar docking has been performed with d(5′-GCTGCGTTAACGCAGC-3′)2 to get initial TTAA-(DB1003)2 and TTAA-(DB2868)2 complexes. The AMBER16 package was used to equilibrate the TTAA-ligand complexe system using OL15 force field modifications for DNA. All MD simulations were performed in explicit solvation conditions where DNA–ligand complexes were solvated in a 72 Å × 72 Å × 72 Å truncated octahedron box filled with approximately 5000 TIP3P water^116^ molecules by using the *TLeap*(117) program in AMBER16. We used 150 mM Na^+^ and Cl^–^ to reach excess salt concentrations, and an appropriate number of ions were added to the systems. This is a salt concentration higher than that required to achieve electrical neutrality but is more biologically relevant. The particle mesh Ewald (PME)^118^ method was used to handle Coulombic interactions, and a 10 Å cutoff was applied to all van der Waals interactions. The MD simulations were carried out using the Sander module with the SHAKE^119^ algorithm applied to constrain all bonds involving hydrogen atoms with an integration time step of 2 fs. In the multistage equilibration protocol, the total system was relaxed with 500 steps of steepest-descent energy minimization. The temperature of the whole system was then gradually increased from 0 to 310 K for over 10 ps under constant-volume conditions. In the final step, the production runs on the system were subsequently performed for 1 μs under *NPT* (constant-pressure) conditions on the PMEMD CUDA module of AMBER16^110^ trajectories and were postprocessed using the CPPTRAJ module of AMBERTOOLS 16^117^ to produce 25,000 snapshots for analysis and visualization in UCSF Chimera visualization software.^120^ The steepest descent algorithm is useful for quickly removing the largest strains in the system but also converges slowly when close to a minimum.

## Data Availability

Data has been deposited in the Protein Data Bank (https://www.rcsb.org/) under the accession numbers PDB IDs 8V4T and 8VIU.

## Supporting Information Available

Synthetic scheme of DB2868, detailed experimental methods for biosensor-SPR, CD competition ESI-MS, and molecular curvature determination (PDF)
