# Supplementary material for: Alternative Approach to Sequence-Specific Recognition of DNA: Cooperative Stacking of Dication Dimers—Sensitivity to Compound Curvature, Aromatic Structure, and DNA Sequence
Source: ACS Chem Biol. 2025 Feb 7;20(2):489–506. doi: 10.1021/acschembio.4c00800 (PMC11851451; doi:10.1021/acschembio.4c00800)
Supplement: Supplementary file 1 — cb4c00800_si_001.pdf [file cb4c00800_si_001.pdf]

An Alternative Approach to Sequence-specific Recognition of DNA: Cooperative Stacking of Dication Dimers-Sensitivity to Compound Curvature, Aromatic Structure, and DNA Sequence

Ananya Paul,[†1] J Ross Terrell,[†1] Abdelbasset A Farahat,[1,2,3] Edwin N Ogbonna,[1] Arvind Kumar,[1] David W Boykin,[1] Stephen Neidle,[*4] W David Wilson*[1]

[1]Department of Chemistry and Center for Diagnostics and Therapeutics Georgia State University, Atlanta, GA 30303, USA

[2]Department of Pharmaceutical Organic Chemistry, Faculty of Pharmacy, Mansoura University, Mansoura 35516, Egypt

[3]Master of Pharmaceutical Sciences Program, California North State University, Elk Grove, California 95757, United States

[4]School of Pharmacy, University College London, London WC1N 1AX, United Kingdom.

[†] These authors contributed equally to this work.

Address correspondence to either of these authors

**W. David Wilson; Email: wdw@gsu.edu*

**Stephen Neidle; Email: s.neidle@ucl.ac.uk*

Chemistry:

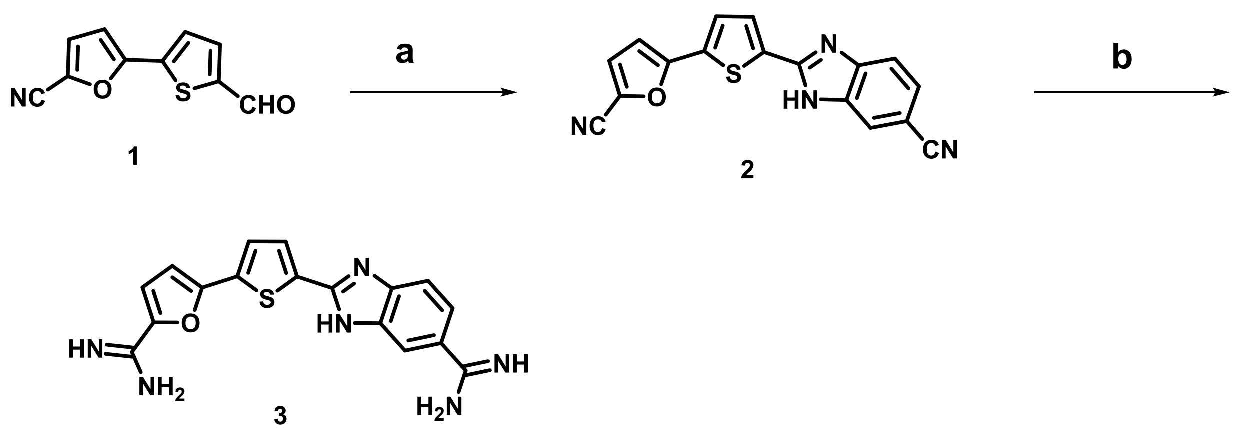

DB 2868

Scheme 1: Reagents and conditions; (a) 3,4-diaminobenzonitrile, $Na_2S_2O_5$, DMSO; (b) i- $NH_2OH.HCl$, Potassium ter butoxide, DMSO, ii- AC_2O,gl. Acetic acid, iii- H2, Pd(C)/EtOH-acetic acid, iv- NaOH sol., HCl gas/EtOH.

The synthesis for previously reported diamidines are provided in the references cited: DB1003, DB1871, DB1992, DB1896, DB2009, DB1898, and DB1897 (1).

Biophysical Experimental:

Materials and sample preparation:

In the circular dichroism (CD) experiments and competition electrospray ionization mass spectrometry (ESI-MS), hairpin oligomer sequences were used (Scheme 1b). In biosensor-surface plasmon resonance (SPR) experiments, the same sequences were used but with a 5′-biotin-label. The DNA oligomers were purchased from Integrated DNA Technologies, Inc. (IDT, Coralville, IA) and were purified using reverse-phase HPLC and characterized by mass spectrometry. The buffer used in the Circular Dichroism (CD) experiment consisted of 50 mM Tris-HCl, 100 mM NaCl, 1 mM EDTA, and had a pH of 7.4 (TNE 100). The biosensor-surface plasmon resonance (SPR) experiments were conducted in filtered and degassed buffer with TNE 100, 0.05% (v/v) surfactant P20.

Biosensor-Surface Plasmon Resonance (SPR)

The SPR measurements were conducted using a four-channel Biacore T200 optical biosensor system (Cytiva, Global life science solutions USA LLC). A streptavidin-derivatized CM5 sensor chip was prepared for use by conditioning it with a series of 180-second injections of 1 M NaCl in 50 mM NaOH (activation buffer), followed by thorough washing with HBS buffer (10 mM HEPES, 150 mM NaCl, 3 mM EDTA, and 0.05% P20, pH 7.4). Biotinylated DNA samples (**AATT**: 5'-biotin-CGA**AATT**GCCTCTGCAATTCG-3'; **TTAA**: 5'-biotin-CG**TTAA**GCTTTCTTAACG-3'; **TATA**: 5'-biotin-CC**TATA**GTTTTCTATAGG-3';) were then used for the coupling with the streptavidin-derivatized surface. The 25-30 nM DNAs were prepared in HBS buffer and immobilized on the flow cell surface through noncovalent capture. desired RU was reached. Flow cell 1 was chosen to left blank as a reference. Flow cells 2 to 4 were individually immobilized by manually injecting biotinylated DNA stock solutions at a flow rate of 1 µL/min until the desired amount of DNA Response Units (RU) was reached (150 to 250 RU). All the ligand solutions were prepared with degassed and filtered TNE 100 with 0.05% (v/v) surfactant P20 by serial dilutions from a concentrated 2 mM DMSO stock solution. A series of different concentrations of each ligand (ranging from 2 nM to 1000 nM) were injected over the DNA sensor chip at a flow rate of 100 µL/min for 180 seconds, followed by buffer flow for ligand dissociation (600 to 1800 seconds). After each cycle, each sensor chip surface was regenerated with a 10 mM glycine solution (pH 2.5) for 30 seconds, followed by multiple buffer injections to achieve a stable baseline for the following cycles. The reference response from the blank cell was subtracted from the response in each flow cell containing DNA to get a signal (RU_{obs}, response units) directly proportional to the amount of bound compound. The predicted maximum response per bound compound in the steady-state region (RU_{max}) was determined from the DNA molecular weight, the amount of DNA on the flow cell, the compound molecular weight, and the refractive index gradient ratio of each compound and DNA. RU_{obs} was plotted as a function of free ligand concentration (C_{free}), and the equilibrium binding constants (K_A) were determined either with a one-site binding model ($K_2 = 0$) (for 1:1 ligand and DNA binding ratio) or with a two-site model (for 2:1 ligand and DNA binding ratio), where $r = (RU_{obs}/RU_{max})$ were representing the moles of bound

compound/mol of DNA hairpin duplex and K_1 and K_2 represent macroscopic binding constants.

$r = (K_1 \cdot C_{free} + 2K_1 \cdot K_2 \cdot C_{free}^2)/(1 + K_1 \cdot C_{free} + K_1 \cdot K_2 \cdot C_{free}^2)$ ………………(1)

The value of RU_{max} can be used as a fitting parameter, and it can be compared to the predicted maximal response per bound ligand in order to independently evaluate the stoichiometry. Kinetic analyses were conducted by globally fitting the binding results for the entire concentration series using a standard 1:1 kinetic model (Biacore Evaluation Software) with integrated mass transport-limited binding parameters, as previously described (2-4).

Circular Dichroism (CD):

Circular dichroism experiments were performed on a Jasco J-1500 CD spectrometer in a 1 cm quartz cuvette at 25 °C. A buffer scan as a baseline was collected first in the same cuvette and subtracted from the scan of the following samples. Circular dichroism experiments were conducted by using Jasco J-1500 CD spectrometer in a 1 cm quartz cuvette at 25°C. First, a baseline buffer scan was performed in the same cuvette and then subtracted from the scan of the subsequent samples. The hairpin DNA sequence -**AATT-** (5 µM) and **-TTAA-** (5 µM), Scheme 1b, in TNE 100, was added to the cuvette before the titration. Then, the ligand was added to the DNA solution and incubated for 15 minutes to allow the DNA-ligand complex to form and reach equilibrium. Four spectra were averaged from 600 to 220 nm wavelength for each titration point using a scan speed of 100 nm/min and a response time of 1 s. Baseline-subtracted graphs were then created using KaleidaGraph 4.0 software.

Competition Electrospray Ionization Mass Spectrometry (ESI-MS):

Electrospray ionization mass spectrometry (ESI-MS) analyses were performed on a Waters Q-TOF micro–Mass Spectrometer (Waters Corporate, Milford, MA) equipped with an electrospray ionization source (ESI) in a negative ion mode. (4) DNA sequences **AATT**: 5'-CGA**AATT**GCCTCTGCAATTCG-3', **TTAA**: 5'-CG**TTAA**GCTTTCTTAACG-3' and **TATA**: 5'-CC**TATA**GTTTTCTATAGG-3' for ESI-MS experiments were purified by dialyzing in 50 mM ammonium acetate buffer (pH 6.7) at 4

ºC with 3x buffer exchange. Test samples were prepared in 100 mM ammonium acetate with 10% v/v methanol at pH 6.7 and introduced into the ion source through a direct infusion at 5 µl/min flow rate. The competitive experiments were done by mixing a ligand and DNAs with different sequences at different ratios. The instrument parameters were typically as follows: capillary voltage of 2800 V, sample cone voltage of 30 V, extraction cone voltage of 1.0 V, desolvation temperature of 70 °C, and source temperature of 100 °C. Nitrogen was used to nebulize and dry gas. Multiply charged spectra were acquired through a full scan analysis at mass range from 300-2500 Da and then deconvoluted to the spectra presented. MassLynx 4.1 software was used for data acquisition and deconvolution.(5)

Molecular Curvature Determination: Method Description:

To determine comparative molecular curvature values for the compounds, a reference circle that passes through both amidine carbons is first defined (Figure S1). These two points are then connected with the reference circle, which has a radius that allows them to pass as closely as possible through the center of each molecular unit of the entire molecule and the two amidine carbons. As the angle of curvature approaches low values, the compounds are quite curved while higher numbers, approaching 180 are more linear. A value of around 140 is ideal for recognition of the minor groove curvature based on binding constants versus relative curvature values (6).

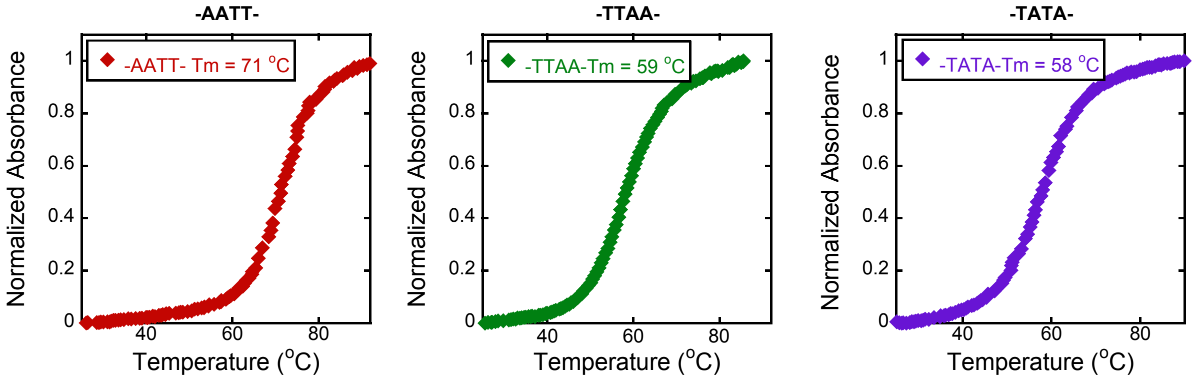

Figure S1: UV melting profiles at 260nm of -AATT-, -TTAA-, and -TATA- DNA sequences. The concentration of each hairpin DNA sequence was 3 μM, and experiments were in TNE100 buffer. The listed values are for an average of two independent experiments with reproducibility of ±0.5 °C.

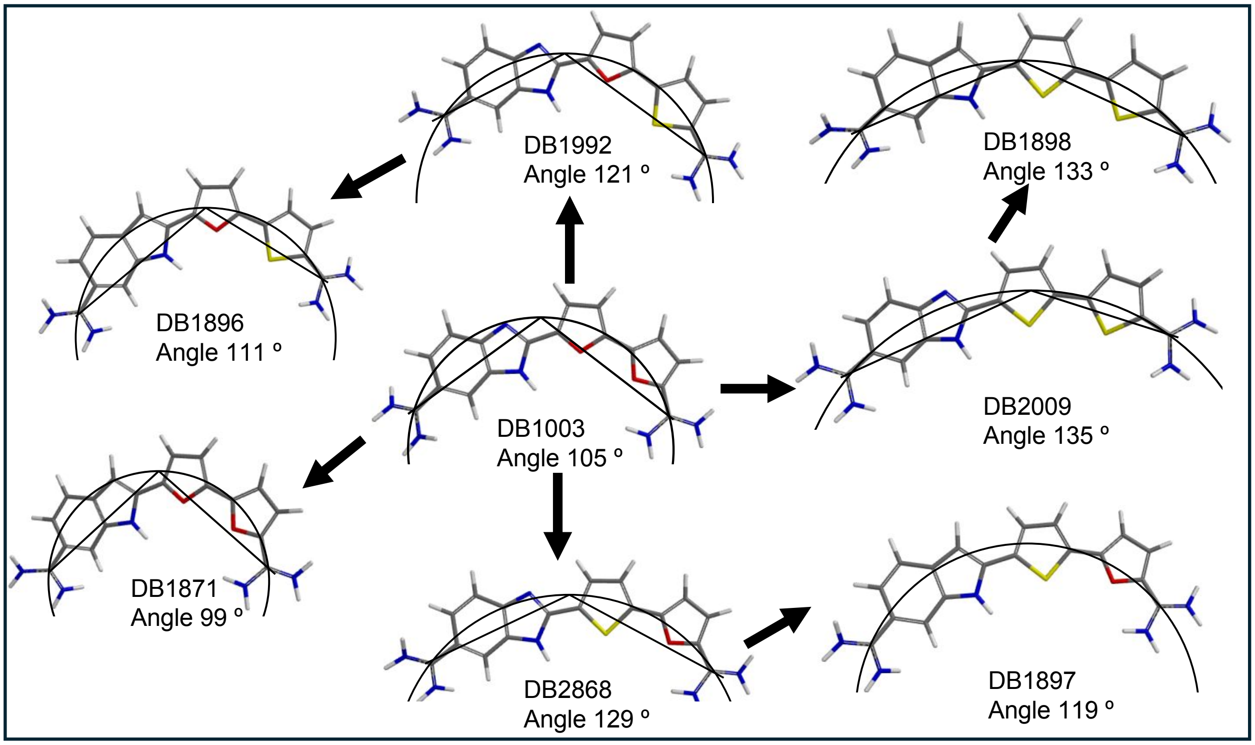

Figure S2: Molecular curvature of the heterocyclic diamidines used in this study. The geometry-minimized structures are obtained from Spartan'20 at B3LYP /6-31G* (p,d) level of theory.

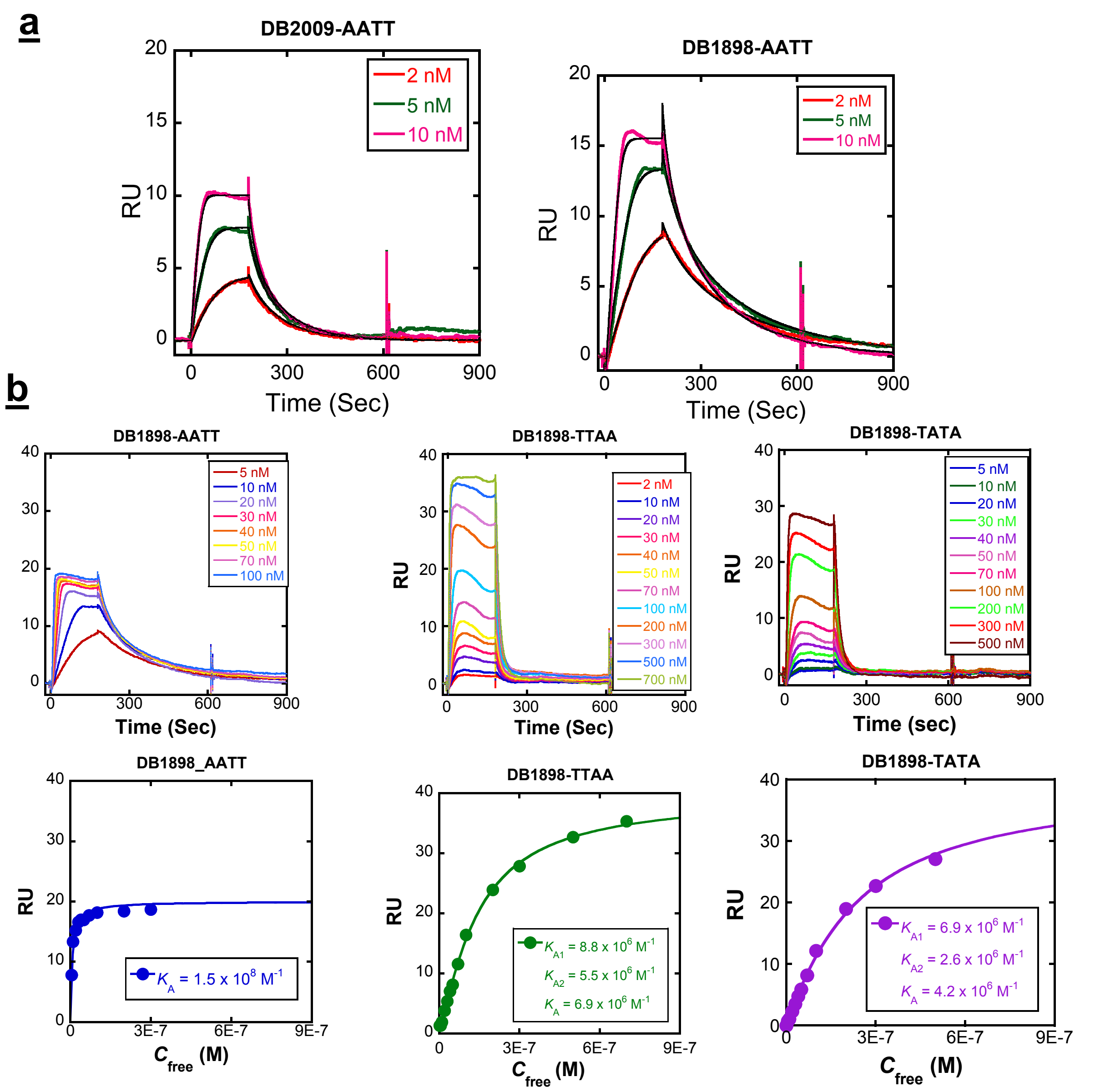

Figure S3: (a) SPR sensorgrams and global kinetic fits (black overlays) for DB2009 with the -AATT- DNA hairpin sequence; The kinetic fit obtained by 1:1 global kinetic fitting; (b) SPR sensorgrams and SPR steady-state affinity binding curves for DNA sequences with diamidines (Scheme 1b) for the interaction of DNA sequences (-AATT-, -TTAA-, and -TATA-). The injected concentrations of each ligand are 2, 5, 10, 15, 20, 30, 40, 50, 70, 100, 200, 300, 500, and 1000 nM in the 50 mM Tris-HCl, 100 mM NaCl,

1 mM EDTA buffer, pH 7.4. In affinity fittings, the lines are the best fit values of a single-site or two-site interaction models, and *K* values are in Table 1 and Table 1.

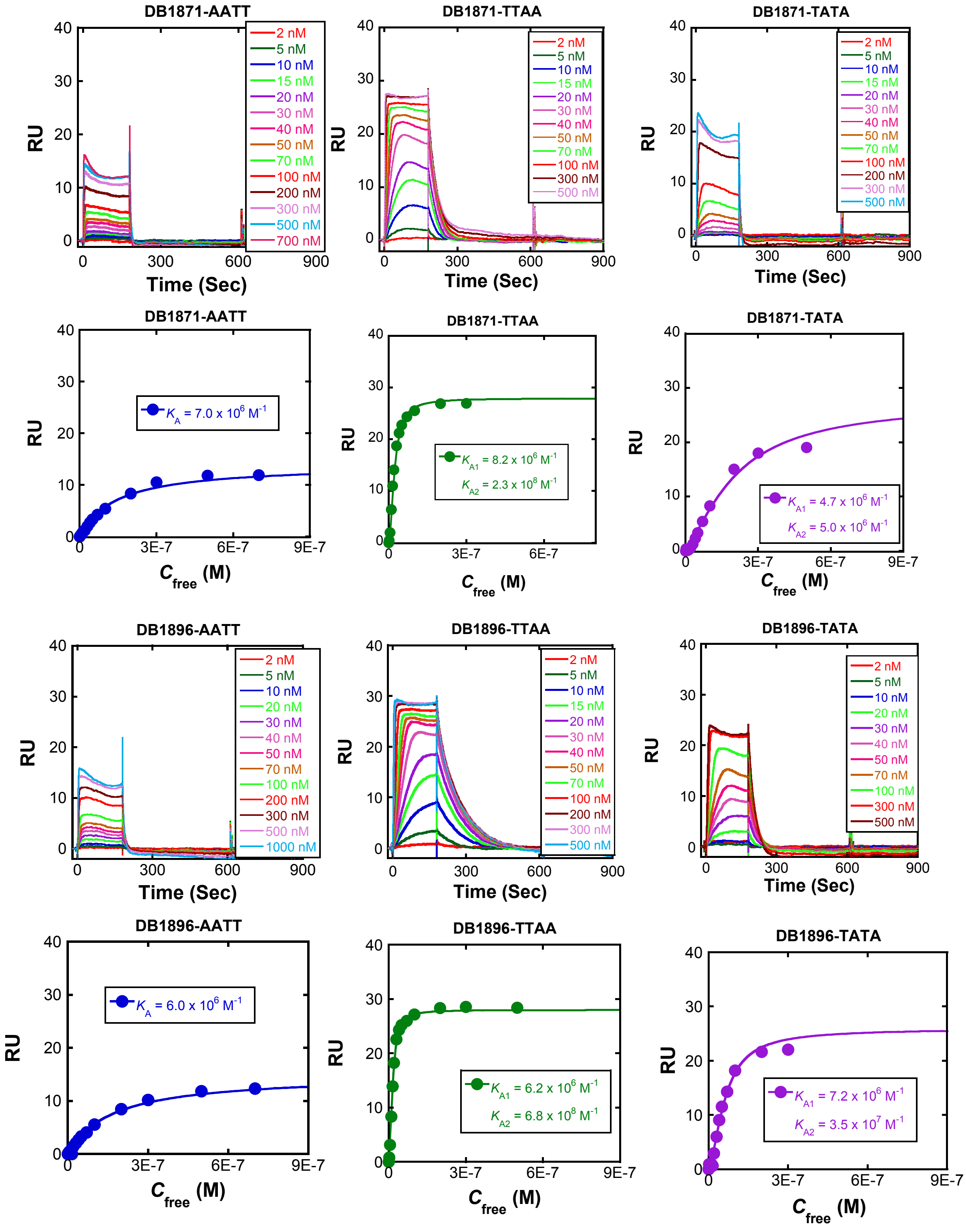
DB1871-AATT
2 nM
5 nM
10 nM
15 nM
20 nM
30 nM
40 nM
50 nM
70 nM
100 nM
200 nM
300 nM
500 nM
700 nM
RU
Time (Sec)
DB1871-TTAA
2 nM
5 nM
10 nM
15 nM
20 nM
30 nM
40 nM
50 nM
70 nM
100 nM
300 nM
500 nM
DB1871-TATA
2 nM
5 nM
10 nM
15 nM
20 nM
30 nM
40 nM
50 nM
70 nM
100 nM
200 nM
300 nM
500 nM
DB1871-AATT
$K_A = 7.0 \times 10^6\ M^{-1}$
C_{free} (M)
DB1871-TTAA
$K_{A1} = 8.2 \times 10^6\ M^{-1}$
$K_{A2} = 2.3 \times 10^8\ M^{-1}$
DB1871-TATA
$K_{A1} = 4.7 \times 10^6\ M^{-1}$
$K_{A2} = 5.0 \times 10^6\ M^{-1}$
DB1896-AATT
2 nM
5 nM
10 nM
20 nM
30 nM
40 nM
50 nM
70 nM
100 nM
200 nM
300 nM
500 nM
1000 nM
DB1896-TTAA
2 nM
5 nM
10 nM
15 nM
20 nM
30 nM
40 nM
50 nM
70 nM
100 nM
200 nM
300 nM
500 nM
DB1896-TATA
2 nM
5 nM
10 nM
20 nM
30 nM
40 nM
50 nM
70 nM
100 nM
300 nM
500 nM
DB1896-AATT
$K_A = 6.0 \times 10^6\ M^{-1}$
DB1896-TTAA
$K_{A1} = 6.2 \times 10^6\ M^{-1}$
$K_{A2} = 6.8 \times 10^8\ M^{-1}$
DB1896-TATA
$K_{A1} = 7.2 \times 10^6\ M^{-1}$
$K_{A2} = 3.5 \times 10^7\ M^{-1}$

Figure S4: SPR sensorgrams and SPR steady-state affinity binding curves for DNA sequences with diamidines (Scheme 1b) for the interaction of DNA sequences (-AATT-, -TTAA-, and -TATA-). The injected concentrations of each ligand are 2, 5, 10, 15, 20, 30, 40, 50, 70, 100, 200, 300, 500, and 1000 nM in the 50 mM Tris-HCl, 100 mM NaCl, 1 mM EDTA buffer, pH 7.4. In affinity fittings, the lines are the best-fit values of a single-site or two-site interaction models, and *K* values are in Table 1.

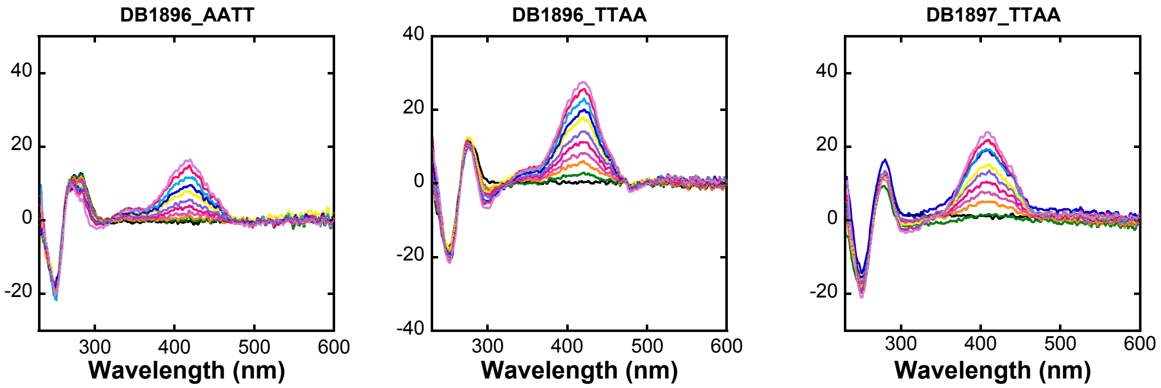

Figure S5: CD titration of -AATT- and -TTAA- DNA sequences with DB1896 and DB1897. The concentration of each duplex hairpin DNA was 5 μM, and each compound was added with 1 μM increments with a total of 10 increments. DB1896 and DB1897 with -TTAA- sequence saturates at 2:1 compound to DNA ratio, confirming dimer formation for these compounds. The experimental buffer condition is Tris-HCl buffer (50 mM Tris-HCl, 100 mM NaCl, 1 mM EDTA, pH 7.4) at 25℃.

Table S1: Crystallographic and refinement statistics

	GCTGCGTTAACGCAGC Native	GCTGCGTTAACGCAGC+DB1992
PDB ID	8V4T	8VIU
Wavelength	0.9793	0.9793
Resolution range	25.74 - 1.47 (1.523 - 1.47)	32.53 - 1.49 (1.543 - 1.49)
Space group	R 3 2 :H	R 3 2 :H
Unit cell	38.324 38.324 163.022 90 90 120	38.345 38.345 162.141 90 90 120
Total reflections	88573 (8506)	85277 (8428)
Unique reflections	8251 (802)	7906 (775)
Multiplicity	10.7 (10.6)	10.8 (10.9)
Completeness (%)	99.56 (100.00)	99.28 (99.87)
Mean I/sigma(I)	23.58 (4.52)	22.13 (4.18)
Wilson B-factor	15.97	16.94
R-merge	0.05398 (0.4983)	0.05583 (0.5523)
R-meas	0.05689 (0.5237)	0.05887 (0.5799)
R-pim	0.01765 (0.1595)	0.01832 (0.1751)
CC1/2	0.999 (0.936)	0.999 (0.947)

CC*	1 (0.983)	1 (0.986)
Reflections used in refinement	8251 (802)	7916 (774)
CC*	820 (80)	784 (77)
R-work	0.1953 (0.2704)	0.2245 (0.2446)
R-free	0.2107 (0.2868)	0.2492 (0.2881)
CC(work)	0.968 (0.867)	0.963 (0.923)
CC(free)	0.957 (0.874)	0.938 (0.845)
Number of non-hydrogen atoms	424	442
macromolecules	325	325
ligands	7	48
solvent	92	69
RMS(bonds)	0.013	0.021
RMS(angles)	1.5	1.69
Ramachandran favored (%)	0	0
Ramachandran allowed (%)	0	0
Ramachandran outliers (%)	0	0
Rotamer outliers (%)	0	0
Clashscore	1.95	1.85
Average B-factor	23.67	25.04
macromolecules		
ligands	22.22	35.57
solvent	30.1	31.33
Number of TLS groups	1	0

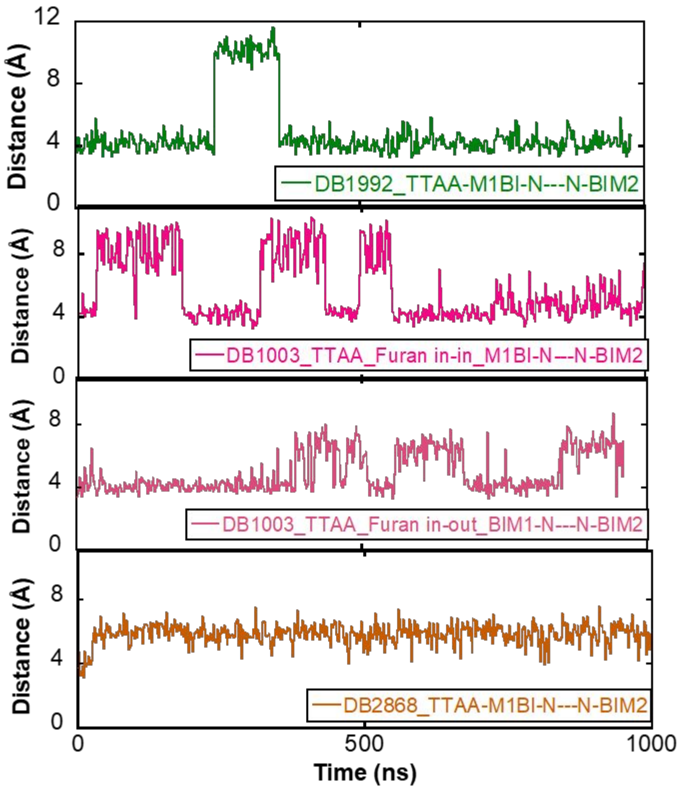

Figure S6: The distance plots between two antiparallel stacked BI-Ns (unprotonated) at the -TTAA-minor groove.

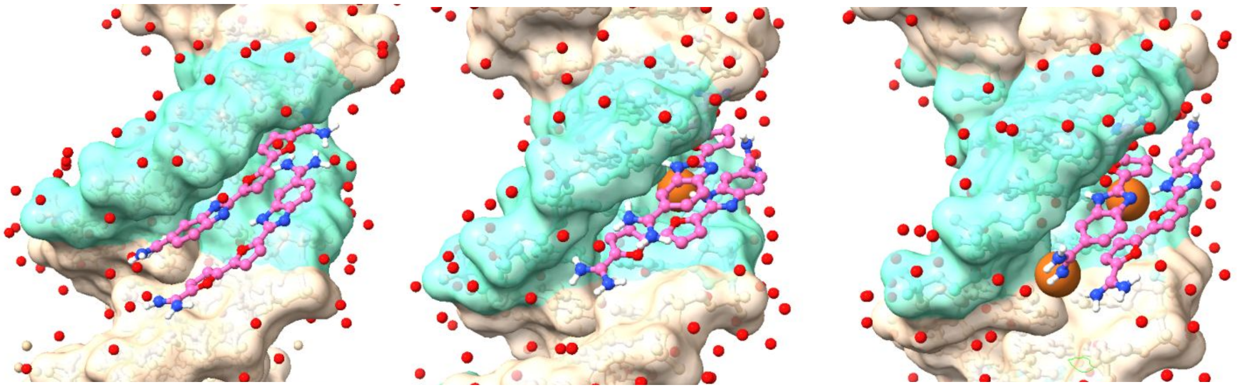

150 ns **300 ns** **800 ns**

Figure S7: A surface model viewed into the minor groove of the -TTAA- binding site with bound stacked DB1003 dimer, where the central furan of oxygen of DB1003 is pointed into the minor groove while the terminal furan oxygen is pointed out of the minor groove. The DNA bases at -TTAA- site are represented in aquamarine color scheme, and the rest of the sequence is represented in tan-color; The stacked DB1003 dimer is in pink-white-red-blue (C–H–O-N) color; The dynamic behavior of stacked DB1003 dimer at the -TTAA- minor groove dimer is illustrated here;

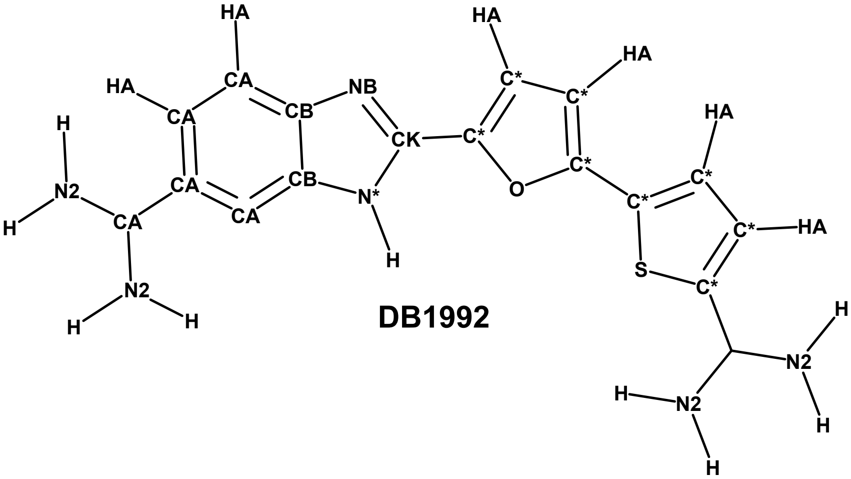

DB1992

Figure S8: Molecular structure with specific atom types used for the DB1992 molecule.

Table S2. Frcmod file of the DB1992 molecule

```
remark goes here #DB1992
MASS
N2 14.01        0.530
CA 12.01        0.360
CB 12.01        0.360
C* 12.01        0.360
CK 12.01        0.360
HA 1.008        0.167
H  1.008        0.161
N* 14.01        0.530
NB 14.01        0.530
S  32.06        2.900
O  16.00        0.465

BOND
CA-CA  469.0    1.400
CA-CB  469.0    1.404
CB-CB  520.0    1.370
CB-N*  436.0    1.374
CB-NB  414.0    1.391
CA-HA  367.0    1.080
CK-N*  440.0    1.371
CK-NB  529.0    1.304
CK-C*  418.3    1.4290
C*-C*  418.3    1.4290
C*-S   279.3    1.7370
C*-HA  347.2    1.0850
CA-N2  481.0    1.340
H -N2  434.0    1.010
H -N*  434.0    1.010
O -C*  376.1    1.3700
CA-C*  411.7    1.4340

ANGLE
CA-CA-CA    63.0     120.00
CA-CA-CB    63.0     120.00
CA-CA-HA    50.0     120.00
CA-CB-CB    63.0     117.30
CB-CA-HA    50.0     120.00
CA-CB-NB    70.0     132.40
CA-CB-N*    70.0     132.40
CB-CB-N*    70.0     106.20
CB-CB-NB    70.0     110.40
N*-CK-NB    70.0     113.90
CB-NB-CK    70.0     103.80
CB-N*-CK    70.0     105.40
C*-CK-NB    67.53    121.69
C*-CK-N*    67.53    121.69
C*-C*-CK    66.24    121.77
```

```
C*-C*-C*    67.880    110.700
CK-N*-H     50.0      128.80
CB-N*-H     50.0      125.80
C*-C*-HA    47.14     120.86
C*-S -C*    41.930    89.910
S -C*-C*    80.780    115.020
CA-CA-N2    70.0      119.99
N2-CA-N2    70.0      120.00
H -N2-H     35.0      120.00
CA-N2-H     50.0      120.00
CK-C*-O     68.39     118.76
C*-O -C*    67.250    106.760
O -C*-C*    69.380    117.090
C*-CA-N2    70.0      119.99
C*-C*-CA    63.0      120.00
S -C*-CA    63.0      120.00
C*-C*-CA    63.0      120.00

DIHE
N2-CA-N2-H     4    9.60      180.0       2.0
H -N2-CA-CA    4    9.60      180.0       2.0
N2-CA-CA-CA    4   -3.118     0.000      -2.0
N2-CA-CA-CA    4    0.609     90.000      1.0
CA-CA-CA-CA    4   14.50      180.0       2.0
CA-CA-CA-HA    4   14.50      180.0       2.0
HA-CA-CA-HA    4   14.50      180.0       2.0
CA-CA-CA-CB    4   14.50      180.0       2.0
CB-CA-CA-HA    4   14.50      180.0       2.0
CA-CA-CB-CB    4   14.00      180.0       2.0
HA-CA-CB-N*    4   14.00      180.0       2.0
HA-CA-CB-NB    4   14.00      180.0       2.0
HA-CA-CB-CB    4   14.00      180.0       2.0
CB-CB-N*-CK    4    6.60      180.0       2.0
CB-CB-N*-H     4    6.60      180.0       2.0
CA-CB-N*-H     4    6.60      180.0       2.0
CA-CB-CB-NB    4   21.80      180.0       2.0
CA-CB-CB-N*    4   21.80      180.0       2.0
CA-CA-CB-NB    4   14.00      180.0       2.
CA-CB-NB-CK    2    5.10      180.0       2.0
CA-CB-N*-CK    4    6.60      180.0       2.
NB-CK-N*-CB    4    6.80      180.0       2.0
N*-CK-NB-CB    2   20.00      180.0       2.0
C*-CK-NB-CB    2   20.00      180.0       2.0
C*-CK-NB-CB    2   20.00      180.0       2.
NB-CK-C*-O     4   -0.6       180.0      -4.0
NB-CK-C*-O     4    3.1       180.0      -2.0
NB-CK-C*-O     4   -0.7       360.0       1.0
CB-N*-CK-C*    4    6.80      180.0       2.0
NB-CK-C*-C*    4    3.1       180.0      -2.0
NB-CK-C*-C*    4   -0.6       180.0      -4.0
NB-CK-C*-C*    4   -0.7       360.0       1.0
```

```
N*-CK-C*-C*    4    3.42        180.0             2.0
N*-CK-C*-O     4   -0.6         180.0            -4.0
N*-CK-C*-O     4    3.1         180.0            -2.0
N*-CK-C*-O     4   -0.7         360.0             1.0
H-N*-CK-C*     4    6.80        180.0             2.0
NB-CK-N*-H     4    6.800       180.0             2.0
CK-C*-C*-C*    4   16.000       180.000           2.0
CK-C*-C*-HA    4   16.000       180.000           2.0
C*-C*-C*-HA    4   16.000       180.000           2.0
O -C*-C*-HA    4   16.000       180.000           2.0
O -C*-C*-C*    4   16.000       180.000           2.0
C*-C*-C*-C*    4   16.000       180.000           2.0
HA-C*-C*-HA    4   16.000       180.000           2.0
C*-O -C*-C*    2    2.100       180.000           2.000
C*-O -C*-CK    2    2.100       180.000           2.000
O -C*-C*-S     4   16.000       180.000           2.0
S -C*-C*-HA    4   16.000       180.000           2.0
S -C*-C*-C*    4   16.000       180.000           2.0
C*-S -C*-C*    1    1.100       180.000           2.000
C*-S -C*-CA    1    1.100       180.000           2.000
S -C*-C*-HA    4   16.000       180.000           2.0
S -C*-CA-N2    4   -3.118       0.000            -2.0
S -C*-CA-N2    4    0.609       90.000            1.0
C*-C*-C*-CA    4   16.000       180.000           2.0
HA-C*-C*-CA    4   16.000       180.000           2.0
C*-C*-CA-N2    4   -3.118       0.000            -2.0
C*-C*-CA-N2    4    0.609       90.000            1.0
C*-CA-N2-H     4    9.60        180.0             2.0
CA-CA-CB-N*    4   14.00        180.0             2.0

IMPROPER
C*-C*-C*-S          1.1         180.0        2.0
C*-CK-C*-O          1.1         180.0        2.0
CB-CK-C*-O          1.1         180.0        2.0
CB-CA-CA-HA         1.1         180.0        2.0
CB-CA-CA-HA         1.1         180.0        2.0
CB-N*-CK-NB         1.1         180.0        2.0
CB-CK-N*-H          1.1         180.0        2.0
CB-CA-CC-N*         1.1         180.0        2.0
CB-CA-CB-NB         1.1         180.0        2.0
CA-CA-CA-CA         1.1         180.0        2.0
CA-N2-CA-N2         1.1         180.0        2.0
CA-C*-C*-S          1.1         180.0        2.0

NONBON
  H          0.6000  0.0157
  HA         1.4590  0.0150
  CA         1.9080  0.0860
  CB         1.9080  0.0860
  CK         1.9080  0.0860
  N*         1.8240  0.1700
```

NB	1.8240	0.1700
N2	1.8240	0.1700
C*	1.9080	0.0860
S	2.0000	0.2500
O	1.6837	0.1700

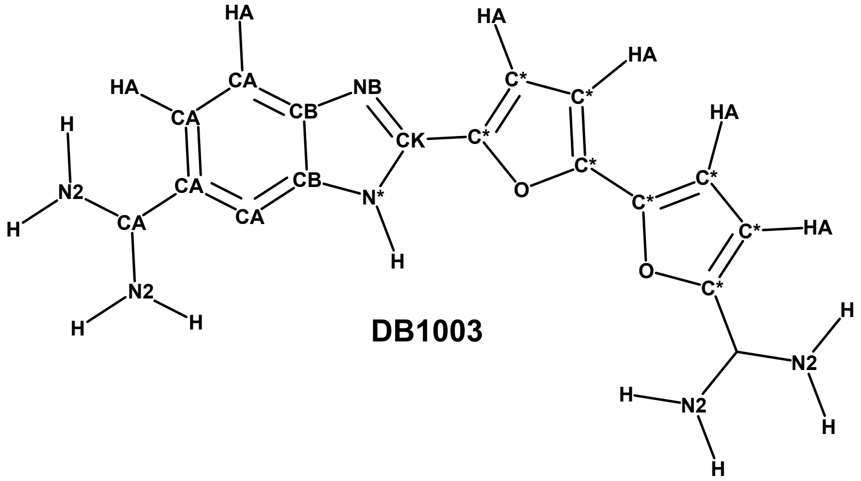

Figure S9: Molecular structure with specific atom types used for the DB1003 molecule.

Table S3. Frcmod file of the DB1003 molecule

```
remark goes here # DB1003
MASS
N2 14.01        0.530
CA 12.01        0.360
CB 12.01        0.360
C* 12.01        0.360
CK 12.01        0.360
HA 1.008        0.167
H  1.008        0.161
N* 14.01        0.530
NB 14.01        0.530
O  16.00        0.465

BOND
CA-CA  469.0   1.400
CA-CB  469.0   1.404
CB-CB  520.0   1.370
CB-N*  436.0   1.374
CB-NB  414.0   1.391
CA-HA  367.0   1.080
CK-N*  440.0   1.371
```

```
CK-NB  529.0    1.304
CK-C*  418.3    1.4290
C*-C*  418.3    1.4290
C*-HA  347.2    1.0850
CA-N2  481.0    1.340
H -N2  434.0    1.010
H -N*  434.0    1.010
O -C*  376.1    1.3700
CA-C*  411.7    1.4340

ANGLE
CA-CA-CA    63.0      120.00
CA-CA-CB    63.0      120.00
CA-CA-HA    50.0      120.00
CA-CB-CB    63.0      117.30
CB-CA-HA    50.0      120.00
CA-CB-NB    70.0      132.40
CA-CB-N*    70.0      132.40
CB-CB-N*    70.0      106.20
CB-CB-NB    70.0      110.40
N*-CK-NB    70.0      113.90
CB-NB-CK    70.0      103.80
CB-N*-CK    70.0      105.40
C*-CK-NB    67.53     121.69
C*-CK-N*    67.53     121.69
C*-C*-CK    66.24     121.77
C*-C*-C*    67.880    110.700
CK-N*-H     50.0      128.80
CB-N*-H     50.0      125.80
C*-C*-HA    47.14     120.86
CA-CA-N2    70.0      119.99
N2-CA-N2    70.0      120.00
H -N2-H     35.0      120.00
CA-N2-H     50.0      120.00
CK-C*-O     68.39     118.76
C*-O -C*    67.250    106.760
O -C*-C*    68.4      117.090
C*-CA-N2    70.0      119.99
C*-C*-CA    63.0      120.00
O -C*-CA    68.4      120.43
C*-C*-CA    63.0      120.00

DIHE
N2-CA-N2-H     4    9.60       180.0         2.
H -N2-CA-CA    4    9.60       180.0         2.0
N2-CA-CA-CA    4   -3.118      0.000        -2.0
N2-CA-CA-CA    4    0.609      90.000        1.0
CA-CA-CA-CA    4   14.50       180.0         2.0
CA-CA-CA-HA    4   14.50       180.0         2.0
HA-CA-CA-HA    4   14.50       180.0         2.0
CA-CA-CA-CB    4   14.50       180.0         2.0
```

CB-CA-CA-HA	4	14.50	180.0	2.0
CA-CA-CB-CB	4	14.00	180.0	2.0
HA-CA-CB-N*	4	14.00	180.0	2.0
HA-CA-CB-NB	4	14.00	180.0	2.0
HA-CA-CB-CB	4	14.00	180.0	2.0
CB-CB-N*-CK	4	6.60	180.0	2.0
CB-CB-N*-H	4	6.60	180.0	2.0
CA-CB-N*-H	4	6.60	180.0	2.0
CA-CB-CB-NB	4	21.80	180.0	2.0
CA-CB-CB-N*	4	21.80	180.0	2.0
CA-CA-CB-NB	4	14.00	180.0	2.
CA-CB-NB-CK	2	5.10	180.0	2.0
CA-CB-N*-CK	4	6.60	180.0	2.
NB-CK-N*-CB	4	6.80	180.0	2.0
N*-CK-NB-CB	2	20.00	180.0	2.0
C*-CK-NB-CB	2	20.00	180.0	2.0
C*-CK-NB-CB	2	20.00	180.0	2.
NB-CK-C*-O	4	-0.6	180.0	-4.0
NB-CK-C*-O	4	3.1	180.0	-2.0
NB-CK-C*-O	4	-0.7	360.0	1.0
CB-N*-CK-C*	4	6.80	180.0	2.0
NB-CK-C*-C*	4	3.1	180.0	-2.0
NB-CK-C*-C*	4	-0.6	180.0	-4.0
NB-CK-C*-C*	4	-0.7	360.0	1.0
N*-CK-C*-C*	4	3.42	180.0	2.0
N*-CK-C*-O	4	-0.6	180.0	-4.0
N*-CK-C*-O	4	3.1	180.0	-2.0
N*-CK-C*-O	4	-0.7	360.0	1.0
H-N*-CK-C*	4	6.80	180.0	2.0
NB-CK-N*-H	4	6.800	180.0	2.0
CK-C*-C*-C*	4	16.000	180.000	2.0
CK-C*-C*-HA	4	16.000	180.000	2.0
C*-C*-C*-HA	4	16.000	180.000	2.0
O -C*-C*-HA	4	16.000	180.000	2.0
O -C*-C*-C*	4	16.000	180.000	2.0
C*-C*-C*-C*	4	16.000	180.000	2.0
HA-C*-C*-HA	4	16.000	180.000	2.0
C*-O -C*-C*	2	2.100	180.000	2.000
C*-O -C*-CK	2	2.100	180.000	2.000
O -C*-C*-O	4	16.000	180.000	2.0
O -C*-C*-HA	4	16.000	180.000	2.0
O -C*-C*-C*	4	16.000	180.000	2.0
C*-O -C*-C*	2	2.100	180.000	2.000
C*-O -C*-CA	2	2.100	180.000	2.000
O -C*-C*-HA	4	16.000	180.000	2.0
O -C*-CA-N2	4	-3.118	0.000	-2.0
O -C*-CA-N2	4	0.609	90.000	1.0
C*-C*-C*-CA	4	16.000	180.000	2.0
HA-C*-C*-CA	4	16.000	180.000	2.0
C*-C*-CA-N2	4	-3.118	0.000	-2.0
C*-C*-CA-N2	4	0.609	90.000	1.0

```
C*-CA-N2-H     4    9.60        180.0            2.0
CA-CA-CB-N*    4   14.00        180.0            2.0

IMPROPER
C*-N*-CK-NB        1.1         180.0        2.0
CA-CB-CB-NB        1.1         180.0        2.0
CA-CA-CB-N*        1.1         180.0        2.0
CB-CA-CA-HA        1.1         180.0        2.0
CA-CA-CA-CA        1.1         180.0        2.0
CB-CA-CA-HA        1.1         180.0        2.0
CA-N2-CA-N2        1.1         180.0        2.0
CB-CK-N*-H         1.1         180.0        2.0
C*-CK-C*-O         1.1         180.0        2.0
C*-C*-C*-O         1.1         180.0        2.0
CA-C*-C*-O         1.1         180.0        2.0

NONBON
  H          0.6000  0.0157
  HA         1.4590  0.0150
  CA         1.9080  0.0860
  CB         1.9080  0.0860
  CK         1.9080  0.0860
  N*         1.8240  0.1700
  NB         1.8240  0.1700
  N2         1.8240  0.1700
  C*         1.9080  0.0860
  O          1.6837  0.1700
```

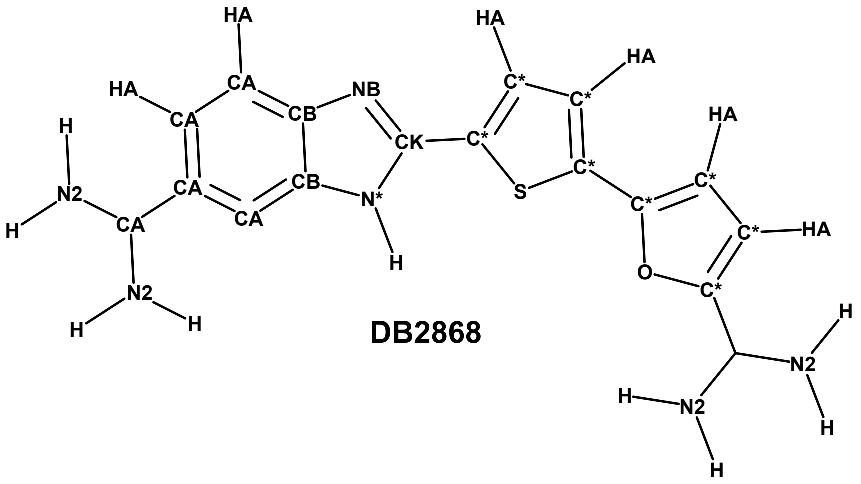

DB2868

Figure S10: Molecular structure with specific atom types used for the DB2868 molecule.

Table S4. Frcmod file of the DB2868 molecule

```
remark goes here #DB2868
MASS
N2 14.01        0.530
CA 12.01        0.360
CB 12.01        0.360
C* 12.01        0.360
CK 12.01        0.360
HA 1.008        0.167
H  1.008        0.161
N* 14.01        0.530
NB 14.01        0.530
O  16.00        0.465
S  32.06        2.900

BOND
CA-CA  469.0    1.400
CA-CB  469.0    1.404
CB-CB  520.0    1.370
CB-N*  436.0    1.374
CB-NB  414.0    1.391
CA-HA  367.0    1.080
CK-N*  440.0    1.371
CK-NB  529.0    1.304
CK-C*  418.3    1.4290
C*-C*  418.3    1.4290
C*-S   279.3    1.7370
C*-HA  347.2    1.0850
CA-N2  481.0    1.340
H -N2  434.0    1.010
H -N*  434.0    1.010
O -C*  376.1    1.3700
CA-C*  411.7    1.4340

ANGLE
CA-CA-CA    63.0     120.00
CA-CA-CB    63.0     120.00
CA-CA-HA    50.0     120.00
CA-CB-CB    63.0     117.30
CB-CA-HA    50.0     120.00
CA-CB-NB    70.0     132.40
CA-CB-N*    70.0     132.40
CB-CB-N*    70.0     106.20
CB-CB-NB    70.0     110.40
N*-CK-NB    70.0     113.90
CB-NB-CK    70.0     103.80
CB-N*-CK    70.0     105.40
C*-CK-NB    67.53    121.69
C*-CK-N*    67.53    121.69
C*-C*-CK    66.24    121.77
```

```
C*-C*-C*    67.880    110.700
CK-N*-H     50.0      128.80
CB-N*-H     50.0      125.80
C*-C*-HA    47.14     120.86
C*-S -C*    41.930    89.910
S -C*-C*    80.780    115.020
CA-CA-N2    70.0      119.99
N2-CA-N2    70.0      120.00
H -N2-H     35.0      120.00
CA-N2-H     50.0      120.00
CK-C*-O     63.0      120.00
C*-O -C*    67.250    106.760
O -C*-C*    69.380    117.090
C*-CA-N2    70.0      119.99
C*-C*-CA    63.0      120.00
O -C*-CA    68.4      120.43
CK-C*-S     80.780    115.020

DIHE
N2-CA-N2-H     4    9.60      180.0       2.0
H -N2-CA-CA    4    9.60      180.0       2.0
N2-CA-CA-CA    4   -3.118     0.000      -2.0
N2-CA-CA-CA    4    0.609     90.000      1.0
CA-CA-CA-CA    4   14.50      180.0       2.0
CA-CA-CA-HA    4   14.50      180.0       2.0
HA-CA-CA-HA    4   14.50      180.0       2.0
CA-CA-CA-CB    4   14.50      180.0       2.0
CB-CA-CA-HA    4   14.50      180.0       2.0
CA-CA-CB-CB    4   14.00      180.0       2.0
HA-CA-CB-N*    4   14.00      180.0       2.0
HA-CA-CB-NB    4   14.00      180.0       2.0
HA-CA-CB-CB    4   14.00      180.0       2.0
CB-CB-N*-CK    4    6.60      180.0       2.0
CB-CB-N*-H     4    6.60      180.0       2.0
CA-CB-N*-H     4    6.60      180.0       2.0
CA-CB-CB-NB    4   21.80      180.0       2.0
CA-CB-CB-N*    4   21.80      180.0       2.0
CA-CA-CB-NB    4   14.00      180.0       2.
CA-CB-NB-CK    2    5.10      180.0       2.0
CA-CB-N*-CK    4    6.60      180.0       2.
NB-CK-N*-CB    4    6.80      180.0       2.0
N*-CK-NB-CB    2   20.00      180.0       2.0
C*-CK-NB-CB    2   20.00      180.0       2.0
C*-CK-NB-CB    2   20.00      180.0       2.
NB-CK-C*-S     4   -0.6       180.0      -4.0
NB-CK-C*-S     4    3.1       180.0      -2.0
NB-CK-C*-S     4   -0.7       360.0       1.0
CB-N*-CK-C*    4    6.80      180.0       2.0
NB-CK-C*-C*    4    3.1       180.0      -2.0
NB-CK-C*-C*    4   -0.6       180.0      -4.0
NB-CK-C*-C*    4   -0.7       360.0       1.0
```

```
N*-CK-C*-C*    4    3.42        180.0             2.0
N*-CK-C*-S     4   -0.6         180.0            -4.0
N*-CK-C*-S     4    3.1         180.0            -2.0
N*-CK-C*-S     4   -0.7         360.0             1.0
H-N*-CK-C*     4    6.80        180.0             2.0
NB-CK-N*-H     4    6.800       180.0             2.0
CK-C*-C*-C*    4   16.000       180.000           2.0
CK-C*-C*-HA    4   16.000       180.000           2.0
C*-C*-C*-HA    4   16.000       180.000           2.0
S -C*-C*-HA    4   16.000       180.000           2.0
S -C*-C*-C*    4   16.000       180.000           2.0
C*-C*-C*-C*    4   16.000       180.000           2.0
HA-C*-C*-HA    4   16.000       180.000           2.0
C*-S -C*-C*    1    1.100       180.000           2.000
C*-O -C*-C*    2    2.100       180.000           2.000
O -C*-C*-S     4   16.000       180.000           2.0
S -C*-C*-HA    4   16.000       180.000           2.0
S -C*-C*-C*    4   16.000       180.000           2.0
C*-S -C*-CA    1    1.100       180.000           2.000
O -C*-C*-HA    4   16.000       180.000           2.0
O -C*-CA-N2    4   -3.118       0.000            -2.0
O -C*-CA-N2    4    0.609       90.000            1.0
C*-C*-C*-CA    4   16.000       180.000           2.0
HA-C*-C*-CA    4   16.000       180.000           2.0
C*-C*-CA-N2    4   -3.118       0.000            -2.0
C*-C*-CA-N2    4    0.609       90.000            1.0
C*-CA-N2-H     4    9.60        180.0             2.0
CA-CA-CB-N*    4   14.00        180.0             2.0
CA-CB-CB-CA    4   21.80        180.0             2.0
CK-C*-S -C*    1    1.100       180.000           2.000
C*-C*-C*-O     4   16.000       180.000           2.0
C*-C*-O -C*    2    2.100       180.000           2.000
C*-O -C*-CA    2    2.100       180.000           2.000

IMPROPER
CB-N*-CK-NB        1.1        180.0        2.0
CA-CB-CB-NB        1.1        180.0        2.0
CA-CA-CB-N*        1.1        180.0        2.0
CA-CA-CA-HA        1.1        180.0        2.0
CA-CA-CA-CA        1.1        180.0        2.0
CA-CA-CA-HA        1.1        180.0        2.0
CA-N2-CA-N2        1.1        180.0        2.0
CA-CK-N*-H         1.1        180.0        2.0
CB-CK-C*-S         1.1        180.0        2.0
C*-C*-C*-S         1.1        180.0        2.0
C*-C*-C*-O         1.1        180.0        2.0
CA-C*-C*-O         1.1        180.0        2.0

NONBON
  H          0.6000  0.0157
  HA         1.4590  0.0150
```

CA	1.9080 0.0860
CB	1.9080 0.0860
CK	1.9080 0.0860
N*	1.8240 0.1700
NB	1.8240 0.1700
N2	1.8240 0.1700
C*	1.9080 0.0860
S	2.0000 0.2500
O	1.6837 0.1700

Figure S11. H[1] NMR spectra for compound DB2868.

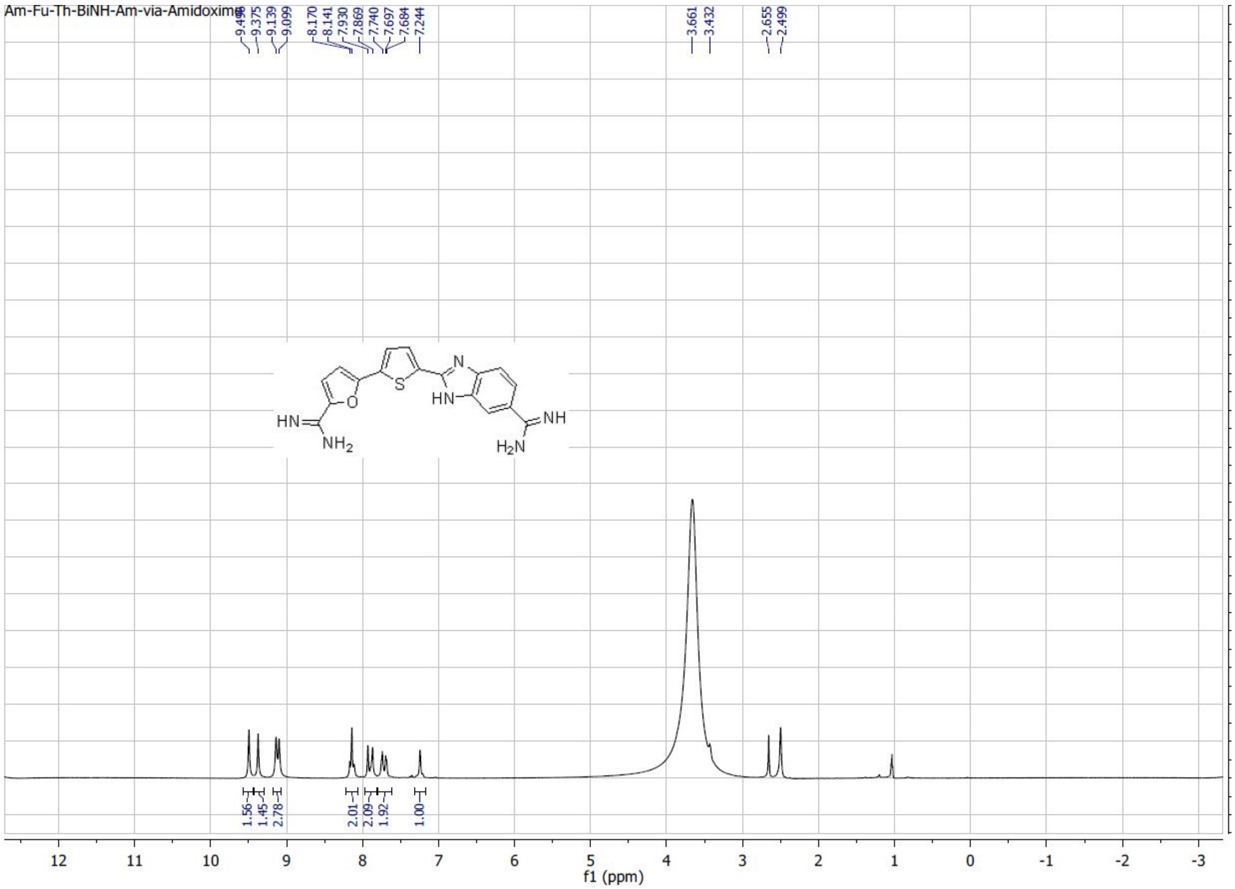

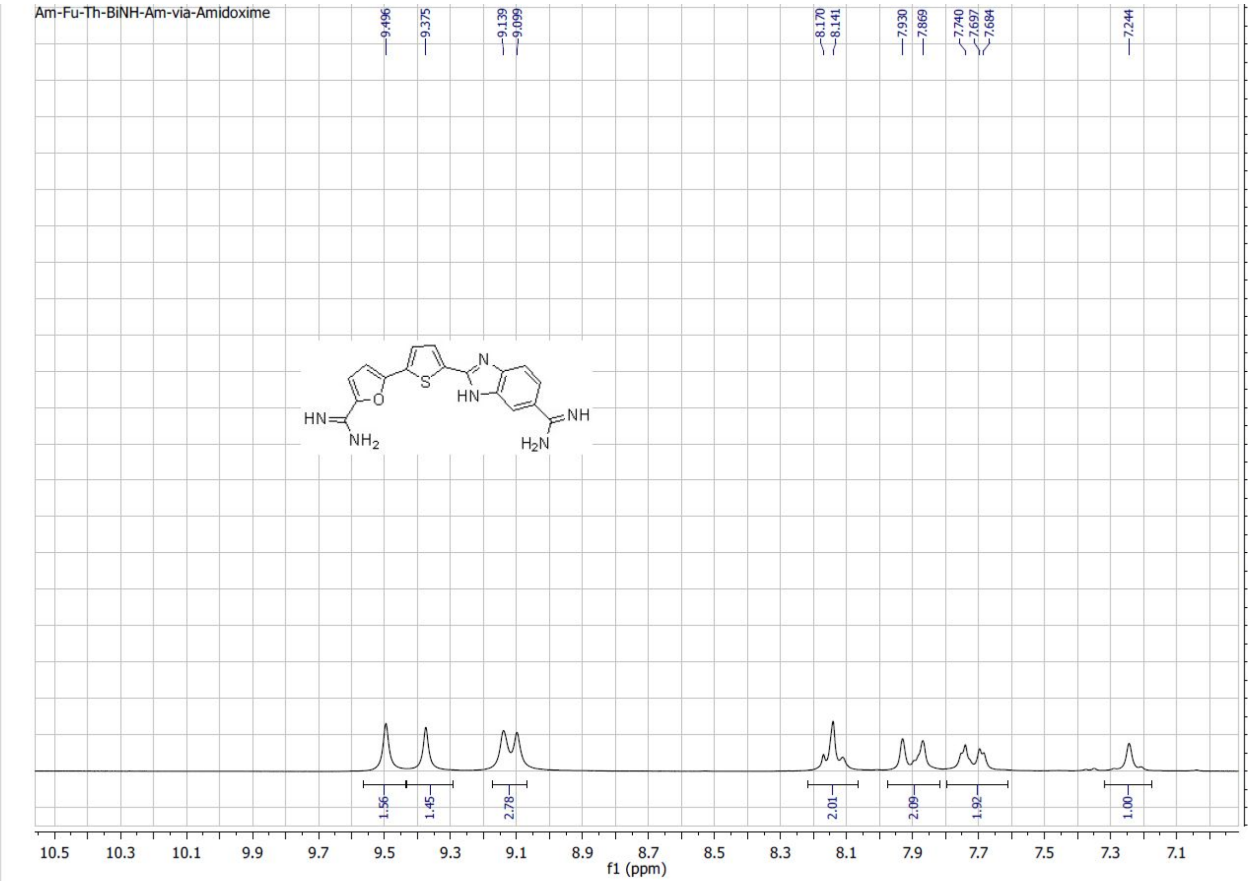

References :

1. Farahat, A. A.; Ismail, M. A.; Kumar, A.; Wenzler, T.; Brun, R.; Paul, A.; Wilson, W. D.; Boykin, D. W. Indole and benzimidazole bichalcophenes: synthesis, DNA binding and antiparasitic activity. *Eur. J. Med. Chem.* **2018**, *143*, 1590-1596.
2. Paul, A.; Musetti, C.; Nanjunda, R.; Wilson, W. D. Biosensor-surface plasmon resonance: label-free method for investigation of small molecule-quadruplex nucleic acid interactions. *Methods*, *Mol. Biol*. **2019**, *2035*, 63-85.
3. Liu, Y.; Chai, Y.; Kumar, A.; Tidwell, R.R.; Boykin, D. W.; Wilson, W. D. Designed compounds for recognition of 10 base pairs of DNA with two at binding sites. *J. Am. Chem. Soc*. **2012**, *134*, 5290-5299.
4. Munde, M.; Kumar, A.; Nhili, R.; Depauw, S.; David-Cordonnier, M.-H.; Ismail, M. A.; Stephens, C. E.; Farahat, A. A.; Batista-Parra, A.; Boykin, D. W.; Wilson, W. D. DNA minor groove induced dimerization of heterocyclic cations: compound structure, binding affinity, and specificity for a TTAA site. *J. Mol. Biol*. **2010**, *402*, 847-864.

5. Laughlin, S.; Wang, S.; Kumar, A.; Boykin, D. W.; Wilson, W. D. A novel approach using electrospray ionization mass spectrometry to study competitive binding of small molecules with mixed DNA sequences. *Anal. Bioanal. Chem*. **2014**, *406*, 6441-6445.
6. Guo, P.; Farahat, A. A.; Paul, A.; Boykin, D. W.; Wilson, W. D. Engineered modular heterocyclic-diamidines for sequence-specific recognition of mixed AT/GC base pairs at the DNA minor groove. *Chem. Sci*. 2021, *2*, 15849-15861.
